# Gut microbiota in acute lung injury/acute respiratory distress syndrome: mechanistic insights and therapeutic opportunities via the gut-lung axis

**DOI:** 10.3389/fcimb.2026.1781229

**Published:** 2026-07-09

**Authors:** Lin Zeng, Bingying Xue, Xintong Ren, Shutong Gong, Jiangtian Yan

**Affiliations:** 1 LiShizhen College of Traditional Chinese Medicine, Huanggang Normal University, Huanggang, Hubei, China; 2 Hubei Key Laboratory of Germplasm Improvement and Utilization of Dabie Shan Dao-di Herbs (Huanggang Normal University), Huanggang, Hubei, China; 3 LiShizhen Culture and Industry Research Center of Traditional Chinese Medicine, Huanggang, Hubei, China

**Keywords:** acute lung injury, acute respiratory distress syndrome, fecal microbiota transplantation, gut microbiota, gut-lung axis, prebiotics, probiotics, traditional Chinese herbal medicines

## Abstract

Acute lung injury (ALI) is a severe clinical syndrome involving inflammatory damage to pulmonary cells, often progressing to acute respiratory distress syndrome (ARDS) with a crude incidence and mortality rate. Despite advances in supportive care, no definitive pharmacological treatment exists. The role of the gut microbiota in immune homeostasis and the gut-lung axis have motivated research into its association with ALI/ARDS. Probiotics, prebiotics, and traditional Chinese herbal medicines have been shown to alleviate ALI/ARDS by modulating gut microbiota. However, the variability among individuals and the complexity of the microbial ecosystem present significant challenges to research and drug development. The integration of multi-omics with artificial intelligence (AI) holds considerable potential for identifying therapeutic targets. This review summarizes the interactions between the gut microbiota and the gut-lung axis in cases of ALI and ARDS. It also discusses the mechanisms through which probiotics, prebiotics, fecal microbiota transplantation (FMT), and traditional Chinese herbal medicines (TCHMs) can intervene, with the aim of developing safer and more effective therapeutic strategies.

## 1 Introduction

Acute lung injury (ALI) is a clinical syndrome characterized by an inflammatory cascade that damages alveolar epithelial cells and capillary endothelial cells. This cascade induces diffuse pulmonary interstitial and alveolar edema, resulting in severe respiratory distress (Wang P. et al., 2025; Zhang Y. et al., 2025). In severe cases, the condition may progress to acute respiratory distress syndrome (ARDS) (Zeng and Yan, 2025a). ARDS is a highly lethal critical illness, with a 40% overall in-hospital mortality rate (rising to 46.1% for severe cases) affecting 10.4% of global intensive care unit patients pre-COVID-19; the pandemic drove a 10-fold increase in U.S. ARDS incidence, with COVID-19-associated ARDS maintaining a persistently high 39% pooled mortality rate (Wick et al., 2024).

Despite significant advancements in supportive therapies for ARDS, a definitive pharmacological treatment for this condition remains elusive (Matthay et al., 2019). Confronted with this unmet clinical need, conventional lung-centric treatment strategies have demonstrated ineffective, prompting researchers to explore the pathological regulation of extrapulmonary organs. Among these, the bidirectional regulatory pathway between the gut and lung, namely the gut-lung axis, has emerged as a pivotal research breakthrough. The gut-lung axis signifies the bidirectional physiological and pathological communication between the intestinal and pulmonary mucosal systems (Yi et al., 2026), underpinned by two core biological foundations. Firstly, the respiratory and gastrointestinal epithelia share a common embryonic origin from the primitive foregut, thereby establishing the intrinsic pathological crosstalk between the two organs (Keely et al., 2012). Secondly, the mucosa-associated lymphoid tissue (MALT) system constitutes the structural basis for systemic immune communication between the gut and lung (Guo J. et al., 2024; Moon et al., 2025). The gastrointestinal tract is home to a highly dynamic microbial ecosystem, known as the gut microbiota. This ecosystem has been identified as a pivotal mediator of gut-lung axis communication (Zhernakova et al., 2016; Budden et al., 2017).

A mounting body of evidence has substantiated the pivotal function of the gut microbiota in preserving immune system balance within the host organism. This has prompted extensive research endeavors in recent years to elucidate the underlying mechanisms by which gut microbiota dysregulation contributes to the development of acute lung injury ALI/ARDS (Pugin and Chevrolet, 1991; Zmora et al., 2019). Beyond the core gut-lung axis, emerging evidence has also revealed that the gut-spleen (Xu et al., 2021), gut-liver (Ran et al., 2026), gut-brain (Chen Q. et al., 2026; Yang et al., 2026), and gut-heart (Chen H. et al., 2026) axes are indirectly involved in pulmonary immune regulation. Together, these axes form the systemic regulatory network of gut microbiota in pulmonary inflammation. In recent years, a substantial body of preclinical research has validated the efficacy of interventions targeting the gut microbiota in alleviating ALI/ARDS. These interventions encompass probiotics, prebiotics, fecal microbiota transplantation (FMT), and traditional Chinese herbal medicines (TCHMs), which have been demonstrated to modulate the gut-lung axis, thereby alleviating lung injury. However, the mechanisms by which these microbiota-targeted interventions alleviate ALI/ARDS remain incompletely elucidated. Three core unresolved scientific questions limit the development and clinical translation of this field. Firstly, does gut microbiota dysbiosis act as a causal driver of ALI/ARDS, or is it merely a secondary consequence of systemic inflammation in critical illness? Secondly, through which cellular and molecular immune circuits do gut-derived microbial signals mediate gut-lung communication to modulate pulmonary immune responses during ALI/ARDS? Thirdly, what are the core translational challenges and clinical application prospects of microbiota-targeted therapies for ALI/ARDS?

This review systematically examines the complex interactions between gut microbiota, microbial metabolites, immune regulation, the gut-lung axis, and ALI/ARDS, in order to address the three core questions previously outlined. The present study aims to elucidate the causal association between gut microbiota dysbiosis and ALI/ARDS. To this end, the study will first clarify the aforementioned association based on clinical and preclinical evidence. Then, the study will proceed to a detailed dissection of the cellular and molecular immune mechanisms of gut-lung communication. We further provide a concise overview of the mechanisms through which probiotics, prebiotics, and TCHMs prevent and treat ALI/ARDS by regulating the gut microbiota and the gut-lung axis. Finally, the discussion encompasses the translational challenges of microbiota-targeted therapies and the potential of integrating multi-omics technologies with artificial intelligence (AI) to identify novel therapeutic targets. The overarching objective of this research is to inform the development of safer and more effective therapeutic strategies for ALI/ARDS.

## 2 Causal association between gut microbiota dysbiosis and ALI/ARDS

The prevailing notion that the lungs are sterile under healthy conditions has been disproven by contemporary metagenomic technologies, which have substantiated the existence of a stable, low-biomass microbial community in the lower respiratory tract that is closely pathologically associated with the gut microbiota (Dickson and Huffnagle, 2015; Ziaka, 2025). The available literature indicates that both clinical patient cohorts and animal model studies have demonstrated that the onset and progression of ALI/ARDS are accompanied by characteristic dysbiosis of the gut and lung microbiota. There is a high degree of correlation between the changes observed in these two microbial communities. In this section, we systematically summarize the characteristic alterations of gut and lung microbiota in ALI/ARDS. We verify the causal relationship between gut microbiota dysbiosis and ALI/ARDS through reverse and forward validation studies. Furthermore, this study distinguished between causal and secondary dysbiosis of the gut microbiota in ALI/ARDS. Finally, we critically evaluate the limitations of existing research.

### 2.1 Characteristic alterations of gut and lung microbiota in ALI/ARDS

#### 2.1.1 Microbiota alterations in clinical patients with ALI/ARDS

A multitude of clinical observational studies have substantiated two assertions. Primarily, patients diagnosed with ARDS manifest gut dysbiosis. Secondly, the prevalence of gut-associated bacteria within the pulmonary microbiota is markedly augmented (**Table 1**) (Dickson et al., 2016; Kyo et al., 2019; Burmeister et al., 2020; Dickson et al., 2020; Li S. et al., 2021; Tang et al., 2022; Hu X. et al., 2023). The prevalence of Enterobacteriaceae, a family of bacteria that colonize the human gut as both commensal bacteria and opportunistic pathogens, is notably elevated in patients with ARDS (Kyo et al., 2019; Dickson et al., 2020). Concurrently, *Bacteroides*, a prevalent commensal genus in the human gut, has been found to be extensively and abundantly present in the lungs of patients with ARDS (Dickson et al., 2016). An additional observational study identified a tendency of elevated bacterial load in the lungs of patients with ARDS (Kyo et al., 2019). This tendency was characterized by a decrease in Betaproteobacteria and an increase in the abundance of Enterobacteriaceae, *Staphylococcu*, and *Streptococcus* (Kyo et al., 2019). These microbial alterations were directly associated with in-hospital mortality (Kyo et al., 2019). Further analysis also revealed elevated levels of Lachnospiraceae in the lung microbiota of ARDS patients, alongside increased levels of Enterobacteriaceae (Dickson et al., 2020).

**Table 1:** Disruption of the lung and/or gut microbiota induced by ALI/ARDS in clinical trials.

The patient’s illness	Patient’s sample	Most relevant lung microbiota	Most relevant gut microbiota	Most relevant molecules/pathways	Ref.
ARDS.	BALF.	*Bacteroides* sp. (OTU009)↑.	Not listed.	Serum: TNF-α.	(Dickson et al., 2016)
ARDS.	BALF.	Shannon index↓. 16S rRNA genes↑. Betaproteobacteria↓; Enterobacteriaceae↑; *Staphylococcu*↑, *Streptococcus*↑.	Not listed.	Serum: IL-6.	(Kyo et al., 2019)
ARDS.	BALF.	16S rRNA genes↑. Enterobacteriaceae↑, Lachnospiraceae↑.	Not listed.	Not listed.	(Dickson et al., 2020)
ARDS.	Fecal.	Not listed.	Firmicutes↑, Clostridiales↑, Ruminococcaceae↑, *Prevotella*↓, *Akkermansia muciniphila*↑, *Eubacterium biforme*↑, *Oxalobacter formigenes*↑, *Ruminococcus flavefaciens*↑.	Not listed.	(Burmeister et al., 2020)
COVID-19.	Fecal.	Not listed.	Number of species↓. Bacteroidetes↑, Firmicutes↓, Erysipelotrichaceae↑, Lachnospiraceae↑, *Lachnospiraceae bacterium* 9143BFAA↓, *Bacteroides faecis*↓, *Bifidobacterium animalis*↑, *Bifidobacterium bifidum*↓, *Clostridium ramosum*↑, *Megasphaera*↓, *Megasphaera* sp.↓, *Parabacteroides goldsteinii*↓, *Paraprevotella*↑, *Paraprevotella* sp.↑,*Roseburia*↓, *Roseburia inulinivorans*↓, *Streptococcus thermophilus*↑.	Butyrate (*Roseburia inulinivorans*↓)	(Li S. et al., 2021)
ALI/ARDS.	Fecal.	Not listed.	Bacteroidetes↑, Firmicutes↓.	Histone metabolites.	(Tang et al., 2022)
AP-ARDS.	Fecal.	Not listed.	Proteobacteria↑, Enterobacteriaceae↑, *Escherichia*-*Shigella*↑, *Bifidobacterium*↓, *Bifidobacterium longum*↓, *Clostridium ramosum*↑, *Klebsiella pneumoniae*↑, *Prevotella copri*↑.	Inflammatory (Proteobacteria↑).	(Hu X. et al., 2023)

↑ mean increase, and ↓ mean decrease.

Concurrent with alterations in the lung microbiota, patients with ARDS and sepsis manifest characteristic gut microbiota dysbiosis, a finding substantiated by large-scale intensive care unit (ICU) cohort studies. Clinical data from patients with ARDS demonstrate distinct shifts in gut microbial composition, characterized by an increase in Bacteroidetes and a decrease in Firmicutes (Tang et al., 2022). In contrast, patients with acute pancreatitis-ARDS (AP-ARDS) exhibit a marked enrichment of Proteobacteria, Enterobacteriaceae, *Escherichia-Shigella*, and *Klebsiella pneumoniae*, accompanied by a reduction in *Bifidobacterium* (including *Bifidobacterium longum*) (Hu X. et al., 2023). It is noteworthy that patients with severe acute respiratory syndrome coronavirus 2 (SARS-CoV-2) infection, a condition known as coronavirus disease 2019 (Covid-19), often exhibit a condition known as ARDS. These patients frequently display reduced gut microbial diversity, increased Bacteroidetes, decreased Firmicutes, and depleted SCFA-producing bacteria such as *Roseburia* (including *Roseburia inulinivorans*), leading to reduced butyrate production (Li S. et al., 2021). Other alterations in the gut microbiota associated with ARDS include an increase in *Akkermansia muciniphila*, *Eubacterium biforme*, *Oxalobacter formigenes*, and *Ruminococcus flavefaciens* (Burmeister et al., 2020). These changes may be a compensatory response to the underlying injury (Burmeister et al., 2020). The α-diversity (Shannon index and Chao1 index) of the gut microbiota in sepsis patients is significantly negatively correlated with the severity of the disease (Long et al., 2023; Sankar et al., 2024). Patients present with a pattern of microbiota imbalance characterized by a decrease in the abundance of beneficial bacteria (*Bifidobacterium*, *Lactobacillus*, *Faecalibacterium prausnitzii*) and the proliferation of pathogenic bacteria (*Escherichia coli*, *Klebsiella pneumoniae*) (Long et al., 2023; Sankar et al., 2024). Longitudinal clinical monitoring data demonstrate that for every one-log increase in the relative abundance of Enterobacteriaceae within seven days following admission to the ICU, the 180-day mortality risk of patients increases by 92% (Xu et al., 2019). It is noteworthy that the reduction of short-chain fatty acids (SCFAs)-producing bacteria and the depletion of fecal and plasma SCFAs occur early in the course of sepsis (Nabizadeh et al., 2023; Li Q. et al., 2025). The levels of these metabolites can independently predict 28-day mortality in critically ill patients (Nabizadeh et al., 2023; Li Q. et al., 2025).

#### 2.1.2 Microbiota alterations in animal models of ALI/ARDS

Preclinical studies in animal models of ALI/ARDS have recapitulated and extended these clinical observations (**Table 2**) (Sze et al., 2014; Poroyko et al., 2015; Dickson et al., 2016; Dessein et al., 2020; Jacobs et al., 2020; Wheatley et al., 2020; Goossens et al., 2022; Tian et al., 2022). In animal models of ALI/ARDS, the lung microbiota has been observed to exhibit elevated levels of gut-colonizing taxa, including Bacteroidales, Bacillaceae, Enterobacteriaceae, Lachnospiraceae sp., Pasteurellaceae, Streptococcaceae, *Enterococcus* sp., and *Streptococcus*, in response to various triggers such as lipopolysaccharide (LPS) i.t. or i.p., cecal ligation and puncture (CLP), scald burn injury, *Pseudomonas aeruginosa* infection, and others (Sze et al., 2014; Poroyko et al., 2015; Dickson et al., 2016; Goossens et al., 2022). Concurrently, studies have reported a reduction in Bacteroidota, Firmicutes, Alicyclobacillaceae, and α-diversity, along with an increase in total bacterial 16S rRNA gene copies (Sze et al., 2014; Poroyko et al., 2015; Dickson et al., 2016; Goossens et al., 2022). These microbes, originating from the gastrointestinal tract, predominate within the dysbiotic lung microbiota in injured animals.

**Table 2:** Dysbiosis of the lung and/or gut microbiota induced by ALI/ARDS in rodent experiments.

Induced-model method		Most relevant lung microbiota	Most relevant gut microbiota	Most relevant molecules/pathways	Ref.
C57/Bl6 mice. LPS (2 µg/g) i.t.		No change in total 16S bacterial load.	Total 16S bacterial load↑.	Not listed.	(Sze et al., 2014)
C57BL/6J mice. LPS (0.63 mg/kg) 20-30 μl i.t.		Firmicutes↓, Proteobacteria↑, Alicyclobacillaceae↓, Brucellaceae↑, Xanthomonadaceae↑.	Not listed.	Not listed.	(Poroyko et al., 2015)
C57Bl/6 mice.	CLP.	Bacterial 16S ribosomal RNA-encoding genes↑. Bacteroidales↑, Bacteroidales (OTU008)↑, Lachnospiraceae sp.↑, *Enterococcus* sp.↑	Not listed.	Not listed.	(Dickson et al., 2016)
	LPS (5mg/kg) i.p.	Enterobacteriaceae↑.			
	LPS (10 μg) i.t.	No bacterial (*E. faecalis* or otherwise) growth.			
Balb/cBy mice. Scald burn injury.		Not listed.	Firmicutes (Peptostreptococcaceae↓, *Dialister*↓, *Enterococcus*↑, *Lactobacillus*↑, *Marvinbryantia*↑, vadinBB60↓).	Anti-microbial peptide (AMP).	(Wheatley et al., 2020)
C57BL/6 mice. Non-absorbable antibiotics (vancomycin 0.5 g/L and colistin 0.15 g/L) p.o. for 7 days. *P*. *aeruginosa* strain PAO1 (5.10^6^ CFU) i.n.		Not listed.	Clostridiales↑, Burkholderiaceae↑, Lactobacillaceae↑, Lachnospiraceae↓, Muribaculaceae↓, Prevotellaceae↓.	Antibiotic-related gut dysbiosis induces lung immunodepression and worsens lung infection in mice.	(Dessein et al., 2020)
C57BL/6 mice. Broad-spectrum antibiotics (ampicillin 1.0 g/L; neomycin 1.0 g/L; metronidazole 1.0 g/L, and vancomycin 0.5 g/L) p.o. for 14 days. LPS (1μg or 10μg) 50 μL i.n.		Not listed.	Diversity index↓.	Antibiotic induced disruption of the intestinal microbiota increase LPS-induced lung inflammation (IL-6).	(Jacobs et al., 2020)
SD rats. LPS (10 mg/kg) i.p.		α-diversity↓. *Brevibacterium*↑.	Not listed.	TNF-α, IL-10, NEU%, IL-1β.	(Tian et al., 2022)
C57BL/6J mice. LPS (200 µg/20g) i.t.		Bacteroidota↓, Bacillaceae↑, Pasteurellaceae↑, Streptococcaceae↑, *Muribacter*↑, *Streptococcus*↑.	Small intestine (both jejunum and ileum): Enterobacteriaceae↑, *Escherichia*-*Shigella*↑, Enterococcaceae↑, *Enterococcus*↑. Ileum: Burkholderiaceae↑, Clostridiaceae↑, Eggerthellaceae↓, Christensellaceae↓, Pasteurellaceae↑, Streptococcaceae↑, *CandidatusArthromitus*↑, *Muribacter*↑, *Ralstonia*↑, *Streptococcus*↑.	MMP2, MMP9.	(Goossens et al., 2022)

↑ mean increase, and ↓ mean decrease.

Concurrently, the gut microbiota of model animals manifests substantial dysbiosis, mirroring clinical observations. The LPS challenge has been shown to result in a significant increase in the total gut bacterial load (Sze et al., 2014). Burn injury has been demonstrated to induce shifts in the composition of the gut microbiota, characterized by a decrease in the abundance of Peptostreptococcaceae, *Dialister*, and vadinBB60, and an increase in *Enterococcus*, *Lactobacillus*, and *Marvinbryantia* (Wheatley et al., 2020). Antibiotic-induced dysbiosis prior to lung injury has been shown to suppress Lachnospiraceae, Muribaculaceae, and Prevotellaceae while elevating Clostridiales, Burkholderiaceae, and Lactobacillaceae (Dessein et al., 2020; Jacobs et al., 2020). This has been demonstrated to reduce gut diversity, thereby exacerbating LPS-induced lung inflammation (Dessein et al., 2020; Jacobs et al., 2020). In the context of LPS-induced ALI, the small intestine (jejunum and ileum) exhibits a pronounced proliferation of Enterobacteriaceae, *Escherichia-Shigella*, Enterococcaceae, *Enterococcus*, Burkholderiaceae, Clostridiaceae, Pasteurellaceae, Streptococcaceae, and *Streptococcus*, accompanied by a decline in Eggerthellaceae and Christensellaceae (Goossens et al., 2022). These phylum- and family-level shifts, including an increase in Proteobacteria and a decrease in Firmicutes, closely resemble the microbial signatures observed in human ARDS patients.

### 2.2 Causal verification of gut microbiota dysbiosis in ALI/ARDS

Observational studies establish a correlation between microbiota dysbiosis and ALI/ARDS but cannot resolve causal direction. Causal involvement of gut microbiota dysbiosis in ALI/ARDS initiation and progression is supported by reverse validation (microbiota depletion worsens injury) and forward validation (microbiota reconstitution mitigates injury). To date, research in this area has focused primarily on preclinical animal studies. Building on this foundational research, it is hoped that further clinical studies with a higher level of evidence will be conducted to establish a causal relationship between acute lung injury/acute respiratory distress syndrome and gut dysbiosis.

#### 2.2.1 Reverse validation: gut microbiota depletion exacerbates the severity of ALI/ARDS

Depletion of the gut microbiota in mice using broad-spectrum antibiotics results in a pronounced escalation of *Streptococcus pneumoniae*-induced bacterial dissemination, pulmonary inflammation, lung injury, and mortality (Schuijt et al., 2016). Concurrently, the phagocytic and immunomodulatory functions of alveolar macrophages were significantly impaired, thereby directly confirming that the intact gut microbiota is a necessary condition for maintaining pulmonary innate immune defense (Schuijt et al., 2016). These findings indicate that gut microbiota dysbiosis can act as a causal driver that compromises lung homeostasis and promotes ALI/ARDS.

#### 2.2.2 Forward validation: gut microbiota reconstitution alleviates ALI/ARDS

FMT is the gold−standard method to validate causality. Multiple studies demonstrate that FMT in ALI/ARDS animal models effectively restores gut microbiota homeostasis, significantly reduces pulmonary inflammation and pathological damage, and improves outcomes (**Table 3**) (Dessein et al., 2020; Li et al., 2020; Tang et al., 2021; Wen et al., 2022; Hua et al., 2023; Zhang et al., 2025b).

**Table 3:** FMT used in the treatment of ALI/ARDS.

Names	Induction method of models	Remodeling effect of the most relevant gut microbiota	Remodeling effect of the most relevant lung microbiota	Relevant signaling molecules/pathways of intestinal barrier	Gut microbiota-derived metabolites	Relevant signaling molecules/pathways of inflammatory response	Ref.
FMT.	SD rats. LPS (5 mg/kg) i.p.	Chao index↑, ACE index↑, Shannon index↑, Simpson index↓. Bacteroidetes↑, Actinobacteria↑.	Not listed.	Not listed.	SCFAs: butyrate↓, acetic acid↑, propionic acid↑.	Lung: TGF-β1/Smads/ERK pathways↓.	(Li et al., 2020)
FMT.	C57BL/6 mice. Non-absorbable antibiotics (vancomycin 0.5 g/L and colistin 0.15 g/L) p.o. 7 days. *P*. *aeruginosa* strain PAO1 (5.10^6^ CFU) i.n.	Clostridiales↓, Burkholderiaceae↓, Lactobacillaceae↓, Muribaculaceae↑, Prevotellaceae↑.	Not listed.	Not listed.	Not listed.	Lung: Macrophages↑, NK cells↑.	(Dessein et al., 2020)
FMT.	C57BL/6 mice. Broad-spectrum antibiotics (neomycin 100 mg/kg; ampicillin 100 mg/kg, vancomycin 50 mg/kg, metronidazole 100 mg/kg) p.o. 8 days. LPS (25 mg/kg) i.t. 4 days.	Actinomycetes↑, Bacteroides↑, Firmicutes↑, Proteobacteria↓.	Not listed.	Plasma: LPS↓.	Not listed.	Lung: TLR4/NF-kB pathway↓.	(Tang et al., 2021)
FMT.	C57/BL6 mice. Broad-spectrum antibiotics [vancomycin (0.5 g/liter), Metronidazole (1 g/liter), and ampicillin (1 g/liter)] treated for 21 days, 75 μL 10^8^ CFU/mL (7.5×10^6^ CFU/mice) of *Pseudomonas aeruginosa* (ATCC 15692) i.t.	Acidobacteriota↓, Actinobacteriota↑, Bacteroidota↓, Campilobacterota↑, Cyanobacteria↑, Desulfobacterota↑, Firmicutes↑, Proteobacteria↓, Verrucomicrobiota↓, Lachnospiraceae_NK4A136_group↑, Muribaculaceae↑, *Alistipes*↑, *Bacteroides*↓, *Helicobacter*↑, *Lactobacillus*↓, *Parabacteroides*↓.	Not listed.	Not listed.	Not listed.	Spleen: Treg↑/Th17↓.	(Wen et al., 2022)
FMT from HUC-MSC-treated mice.	C57BL/6J mice. Broad-spectrum antibiotics (0.1 mg/g neomycin, 0.1 mg/g ampicillin, 0.1 mg/g metronidazole, and 0.05 mg/g vancomycin) by gavage twice per day for 8 days, 100 μl LPS (10 mg/kg) i.p.	*Acinetobacter*↑, *Bacteroides*↓, *Christensenella*↓, *Coprococcus*↓, *Roseburia*, *Sutterella*↑, *Xenorhabdus*↑.	Not listed.	Ileal: ZO-1↑, claudin-1↑, occluding↑.	Not listed.	Peripheral blood: CD4^+^ Th↑, CD8a^+^ Tc↑, CD86^+^ DC↑, CD3e^+^ NK↑, CD25^+^ Tregs↓, CD4^+^ Tregs↓. Lung: TLR4/NF-κB pathway↓, Nrf2/HO-1 pathway↑.	(Hua et al., 2023)
FMT.	SD rats. LPS (10 µg LPS dissolved in 50 µL PBS, 10 mg/kg) i.t.	Shannon index↓, Simpson index↑. Muribaculaceae↑, *Adlercreutzia*↑, *Prevotella*↑, *Romboutsia*↓, Clostridia_UCG-014↑.	Not listed.	Not listed.	SCFAs: butyrate metabolic pathways↑.	Small intestine and lung: Treg↑/Th17↓. Lung: JAK/STAT pathway↓.	(Zhang et al., 2025b)

↑ mean increase, and ↓ mean decrease.

At the level of the gut microbiota, FMT has been shown to markedly remodel microbial composition and diversity in models of ALI/ARDS. In rats with LPS-induced sepsis, FMT has been observed to increase the Chao index, ACE index, and Shannon index, while concurrently decreasing the Simpson index (Li et al., 2020). Additionally, FMT has been shown to enhance the abundance of Actinobacteria and Bacteroidetes (Li et al., 2020). In a murine model of *Pseudomonas aeruginosa* or LPS challenge, FMT has been observed to modulate the composition of the gut microbiota, with an increase in Actinomycetes, Bacteroides, and Firmicutes, and a reduction in Proteobacteria (Tang et al., 2021; Wen et al., 2022). The effects of FMT on the gut microbiota have also been shown to include the upregulation of Muribaculaceae, Prevotellaceae, and Clostridia_UCG 014, and the downregulation of Burkholderiaceae, Lactobacillaceae, and *Romboutsia* (Dessein et al., 2020; Zhang et al., 2025b). FMT has been demonstrated to regulate specific genera, including *Acinetobacter*, *Adlercreutzia*, *Alistipes*, *Helicobacter*, and *Parabacteroides*, with the objective of rebuilding gut microbial homeostasis (Wen et al., 2022; Hua et al., 2023; Zhang et al., 2025b).

With respect to the function of the intestinal barrier, FMT has been demonstrated to enhance intestinal integrity through the upregulation of tight junction proteins, including zonula occludens-1 (ZO-1), claudin 1, and occludin, in the ileum (Hua et al., 2023). Additionally, FMT has been shown to reduce plasma LPS levels, thereby constraining endotoxin translocation (Tang et al., 2021). The effects of microbiota-derived metabolites on the composition of SCFAs have been demonstrated. In particular, FMT has been shown to modulate the levels of acetic acid and propionic acid in the SCFAs profile, as well as to alter the levels of butyrate (Li et al., 2020). Furthermore, enhanced butyrate metabolic pathways have been observed in response to FMT (Zhang et al., 2025b). These effects contribute to the mitigation of lung inflammation and injury.

Mechanistically, the protective effect of FMT on ALI/ARDS is achieved through multiple pathways, including the activation of the nuclear factor erythroid 2-related factor 2 (Nrf2)/heme oxygenase 1 (HO-1) pathway (Hua et al., 2023), the transforming growth factor (TGF)-β1/Smads/extracellular signal-regulated kinase (ERK) pathway (Li et al., 2020), and the Toll-like receptor 4 (TLR4)/nuclear factor kappa B (NF-κB) pathway (Tang et al., 2021; Hua et al., 2023). Concurrently, FMT modulates the equilibrium of T helper cell 17 (Th17)/regulatory T cells (Treg) through the Janus kinase (JAK)/signal transducer and activator of transcription (STAT) pathway, thereby reactivating immune responses in the lungs and spleen and effectively ameliorating LPS-induced ARDS (Wen et al., 2022; Zhang et al., 2025a). Furthermore, FMT has been demonstrated to promote the infiltration and activation of pulmonary macrophages and natural killer (NK) cells, thereby enhancing local immune defense against pathogenic infection (Dessein et al., 2020).

Furthermore, the colonization of specific beneficial bacteria (probiotics) has been demonstrated to alleviate ALI/ARDS in various animal models. For instance, *Akkermansia muciniphila*, a pivotal commensal bacterium that modulates the intestinal mucosal barrier, has been shown to markedly ameliorate LPS-induced lung injury by means of enhancing the expression of tight junction proteins, regulating the microbiota structure, and inhibiting the TLR2/MyD88/NF-κB pathway (Shen et al., 2023b). These findings provide further confirmation that modulation of the gut microbiota can directly affect the progression of ALI/ARDS, thereby providing strong forward evidence for the causal role of the gut microbiota.

#### 2.2.3 Contexts of causal versus secondary dysbiosis

Gut microbiota dysbiosis has been identified as a causal driver in ALI/ARDS under specific conditions. These include instances where microbiota depletion precedes lung injury, exacerbating tissue damage. Additionally, when FMT or probiotic administration independently mitigates ALI/ARDS, and when microbial changes precede clinical lung injury and correlate with mortality, it suggests a potential role for gut microbiota dysbiosis in the development of ALI/ARDS. Conversely, when pulmonary inflammation leads to gut microbiota dysbiosis, rather than gut microbiota dysbiosis causing intestinal bacteria to colonize the lungs and trigger inflammation, this is classified as a secondary alteration of the gut microbiota. The underlying cause of ARDS dictates the nature of the condition. For instance, ARDS associated with non-pneumonic sepsis—such as that caused by trauma or other factors—tends to manifest as causal dysbiosis due to the rapid onset of a systemic inflammatory response. Conversely, ARDS associated with pneumonia (or pneumonic sepsis) caused by pathogenic microorganisms such as bacteria and viruses may involve more secondary dysbiosis. In summary, the disruption of the gut microbiota and the subsequent progression of ALI/ARDS may be, to a certain extent, mutually causative, irrespective of the presence of causal or secondary dysbiosis. However, future research has the potential to concentrate on the gut-lung axis-related microbial changes associated with different etiologies, with the objective of developing more refined and targeted therapies.

### 2.3 Critical evaluation and limitations of current studies

Despite the mounting evidence supporting the causal role of gut microbiota dysbiosis in ALI/ARDS, a critical evaluation of the limitations of existing studies is necessary to avoid overinterpretation of the results.

Firstly, it must be acknowledged that clinical observational studies are subject to three key confounding factors that limit the confirmation of causal association. Firstly, broad-spectrum antibiotic exposure, which is universal in critically ill patients, non-selectively disrupts the respiratory and gut microbiota, and is a critical confounding factor for microbiota dysbiosis. Secondly, mechanical ventilation procedures may cause microbial contamination of the lower respiratory tract, leading to false-positive results of gut-associated bacteria enrichment in the lung. Thirdly, the pulmonary inflammatory microenvironment may drive the expansion of translocated gut-associated bacteria, rather than bacterial colonization driving inflammation, indicating the possibility of reverse causality.

Secondly, the translational value of fundamental research is constrained by numerous factors. The preponderance of extant causal verification evidence derives from SPF laboratory mice, while the gut microbiota composition of experimental mice is, in essence, divergent from that of humans. Standardized animal modeling methods are also incapable of fully replicating the heterogeneous pathological process of clinical ARDS patients, resulting in a significant gap in the translation of experimental results to clinical practice. Moreover, the majority of extant studies have predominantly centered on the comprehensive microbiota dysbiosis phenotype, yet have not yet identified the key pathogenic bacteria or core beneficial bacteria that are driving ALI/ARDS. Furthermore, there is a paucity of research distinguishing the differences in microbiota dysbiosis characteristics and mechanisms of action in ARDS caused by different etiologies (sepsis, trauma, viral infection, etc.). This is a key gap in current research.

These limitations highlight the need for longitudinal clinical studies controlling for antibiotic exposure and ventilation, standardized multi-model preclinical designs, and etiology-stratified microbiota profiling to advance translational understanding.

## 3 Core mechanisms of immune communication along the gut-lung axis

The lung is a core mucosal innate immune organ, enriched with professional innate immune cells (alveolar macrophages, AMs; innate lymphoid cells, ILCs; dendritic cells, DCs) and non-professional immune cells (alveolar epithelial cells, AECs; pulmonary vascular endothelial cells) (Bienenstock, 1984). These cells are the primary targets of gut-derived microbial signals, and their functional modulation constitutes the core of gut-lung axis immune communication.

This section focuses on the hierarchical core mechanisms of gut-lung crosstalk in acute lung injury/acute respiratory distress syndrome (ALI/ARDS), prioritizing three key modules: the upstream initiating pathway (direct immune activation by gut microbial structural components), the core regulatory pathway (remote immunomodulation by dominant gut microbial metabolites), and the downstream effector system (critical immune cell circuits mediating gut-lung communication). These modules form an integrated immune regulatory network, through which gut microbial signals modulate pulmonary innate and adaptive immunity to govern the onset, progression and resolution of ALI/ARDS. The limitations of current mechanistic research are also summarized to guide follow-up studies.

### 3.1 Direct immune activation pathway mediated by gut microbial structural components

**Figure 1:**
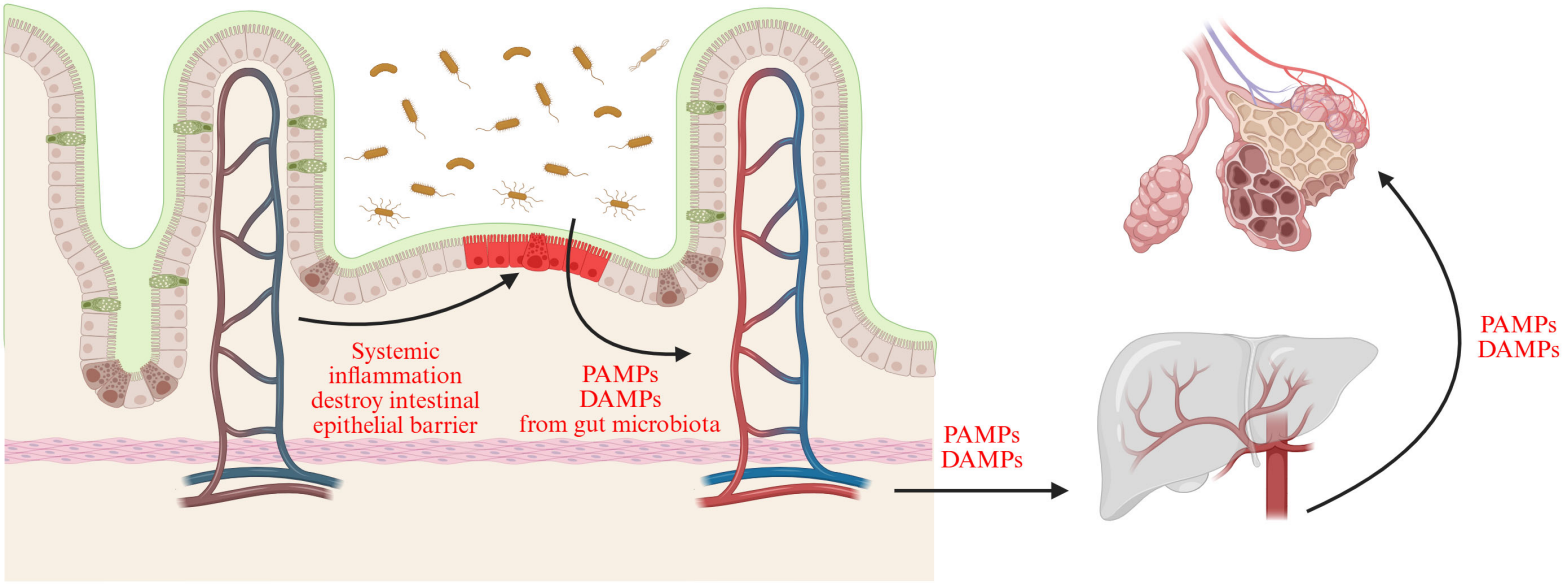
After damage to the intestinal epithelial barrier, PAMPs and DAMPs produced by the disturbed gut microbiota are transported to the lung via the gut-lung axis. DAMPs, damage-associated molecular patterns; PAMPs, pathogen-associated molecular patterns.

The pathway is the fundamental initiating link of gut-lung axis immune disorder in ALI/ARDS. Systemic inflammation and intestinal ischemia-hypoxia impair intestinal epithelial barrier integrity, allowing gut microbial components to translocate and trigger pulmonary inflammatory cascades. Under this condition, pathogen-associated molecular patterns (PAMPs) and damage-associated molecular patterns (DAMPs) from gut microbiota cross the impaired barrier, enter the systemic circulation via the mesenteric lymphatic system and portal vein, and reach the lungs to initiate innate immune responses (Guo J. et al., 2024) (**Figure 1**).

Pattern recognition receptors (PRRs), including Toll-like receptors (TLRs), NOD-like receptors (NLRs) and other major subtypes, are expressed on pulmonary immune cells (Takeuchi and Akira, 2010). As core sensors of gut microbial signals, PRRs specifically recognize conserved PAMPs from translocated gut bacteria, such as LPS from Gram-negative bacteria and peptidoglycan (PGN) from Gram-positive bacteria (Fraiture and Brunner, 2014; Kumar, 2020). For example, Bacteroides-derived LPS in ARDS patients binds to TLR4 on pulmonary immune cells to trigger innate immune activation (Dickson et al., 2016; Kim et al., 2025).

**Figure 2:**
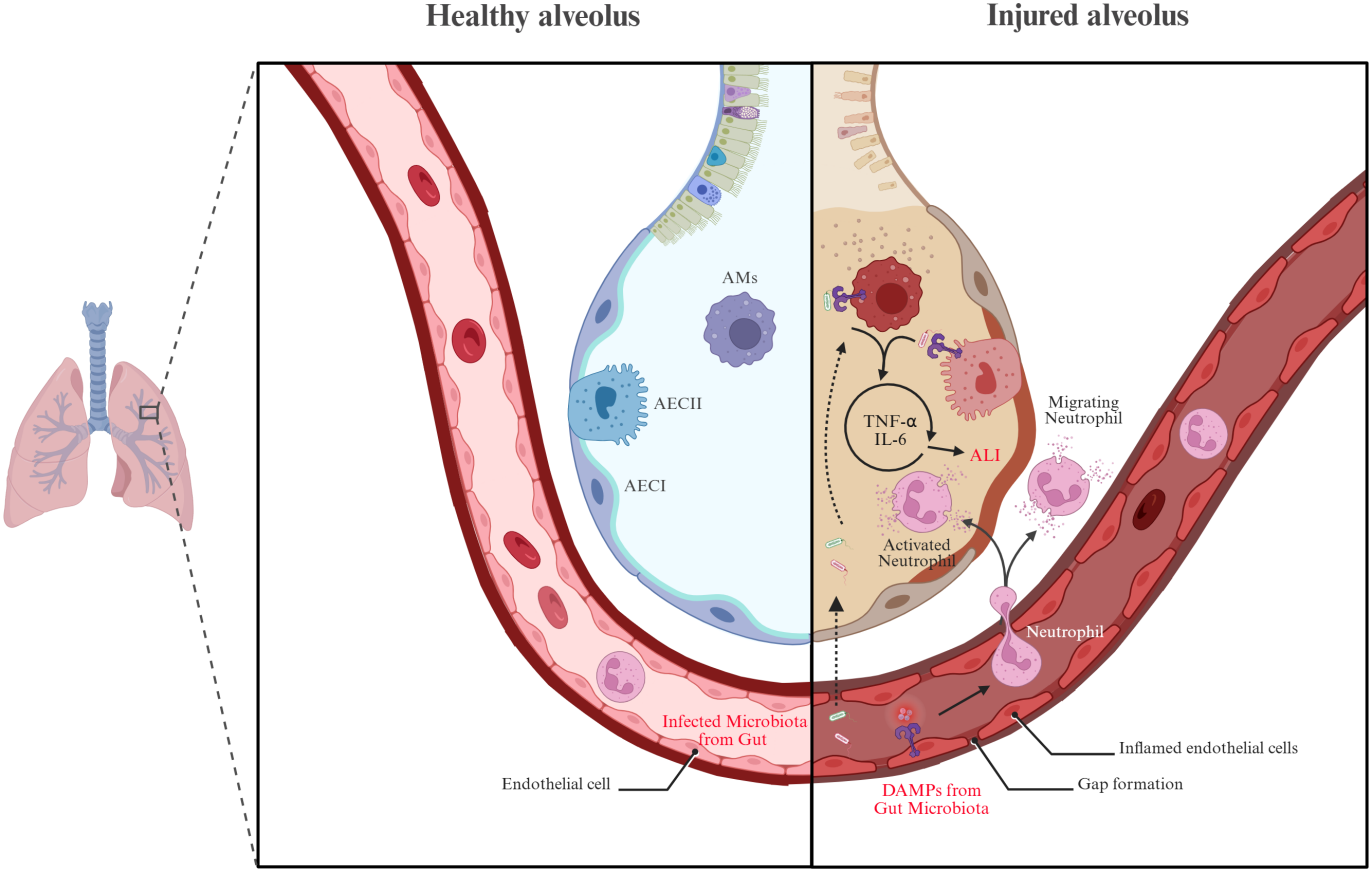
The migration of gut microbiota influences the pathological process of ALI/ARDS. AECs, alveolar epithelial cells; ALI, acute lung injury; AMs, alveolar macrophages; DAMPs, damage-associated molecular patterns; IL, interleukin; TNF, tumor necrosis factor.

Gut barrier damage and dysbiosis also induce the release of DAMPs (e.g., high mobility group box protein B1, HMGB1; extracellular cold-induced RNA-binding protein, eCIRP; adenosine triphosphate, ATP; heat shock proteins, HSPs); and circulating free DNA, cfDNA) (Cicchinelli et al., 2024). Both PAMPs and DAMPs activate intracellular signaling cascades in pulmonary cells, including the NF-κB, mitogen-activated protein kinase (MAPK) and NLRP3 inflammasome pathways, and NOD-like receptor thermal protein domain associated protein 3 (NLRP3) inflammasome pathways (Zeng and Yan, 2025b). These pathways upregulate the transcription of pro-inflammatory cytokines [tumor necrosis factor (TNF)-α, interleukin (IL)-1β, IL-6], drive inflammatory cascades, promote neutrophil infiltration, and ultimately damage the alveolar-capillary membrane—the core pathological feature of ALI/ARDS (Matthay and Zimmerman, 2005; Mason et al., 2017; Zeng and Yan, 2025a) (**Figure 2**).

### 3.2 Remote immunomodulatory pathway mediated by gut microbiota-derived metabolites

Beyond direct structural component activation, gut microbiota exert dominant remote regulatory effects on pulmonary immunity via small-molecule metabolites that enter the systemic circulation. This pathway is the core non-redundant route of gut-lung communication, and this section focuses on the most clinically relevant metabolites with definitive immunomodulatory mechanisms.

#### 3.2.1 SCFAs

Short-chain fatty acids (SCFAs) are the best-characterized functional metabolites in the gut-lung axis, and their immunomodulatory effects run through the entire pathological process of ALI/ARDS. As the main end products of gut commensal bacterial fermentation of dietary fiber, acetate (C2), propionate (C3) and butyrate (C4) account for over 95% of total intestinal SCFAs (Sun et al., 2017). Acetate is produced by a wide range of bacterial groups within the gut microbiota (Louis et al., 2010; Louis et al., 2014; Sun et al., 2017). Propionate is primarily synthesized by Bacteroidetes via the succinate pathway and by Firmicutes via the lactate pathway (Louis et al., 2010; Louis et al., 2014; Sun et al., 2017). Butyrate is chiefly produced by Firmicutes species, including *Faecalibacterium prausnitzii* and *Eubacterium rectale* (Louis et al., 2010; Louis et al., 2014; Sun et al., 2017). With regard to the subject of specific metabolic pathways, acetate, the most abundant SCFAs in the intestinal lumen, is chiefly produced by pyruvate metabolism through the acetyl-coenzyme A (acetyl-CoA) or Wood-Ljungdahl pathways (Ríos-Covián et al., 2016; Cosme et al., 2022). Intestinal bacteria convert monosaccharides degraded from dietary fiber to pyruvate via glycolysis or the pentose phosphate pathways (Ríos-Covián et al., 2016; Cosme et al., 2022). Subsequently, pyruvate is converted to propionate via succinate or acrylate pathways (Ríos-Covián et al., 2016; Cosme et al., 2022). Butyrate, on the other hand, is formed by specialized anaerobic bacteria via acetyl-coenzyme A and butyrate kinase pathways (Ríos-Covián et al., 2016; Cosme et al., 2022). SCFAs (mainly acetic acid) can also be produced via fatty acid oxidation in host cells (endogenous synthetic pathway) (Deleu et al., 2021; Al Mahri et al., 2022). After intestinal absorption, SCFAs enter the systemic circulation and stably reach the lungs to exert biological functions (Boets et al., 2015; Sun et al., 2017; Liu et al., 2021; Lee et al., 2022).

**Figure 3:**
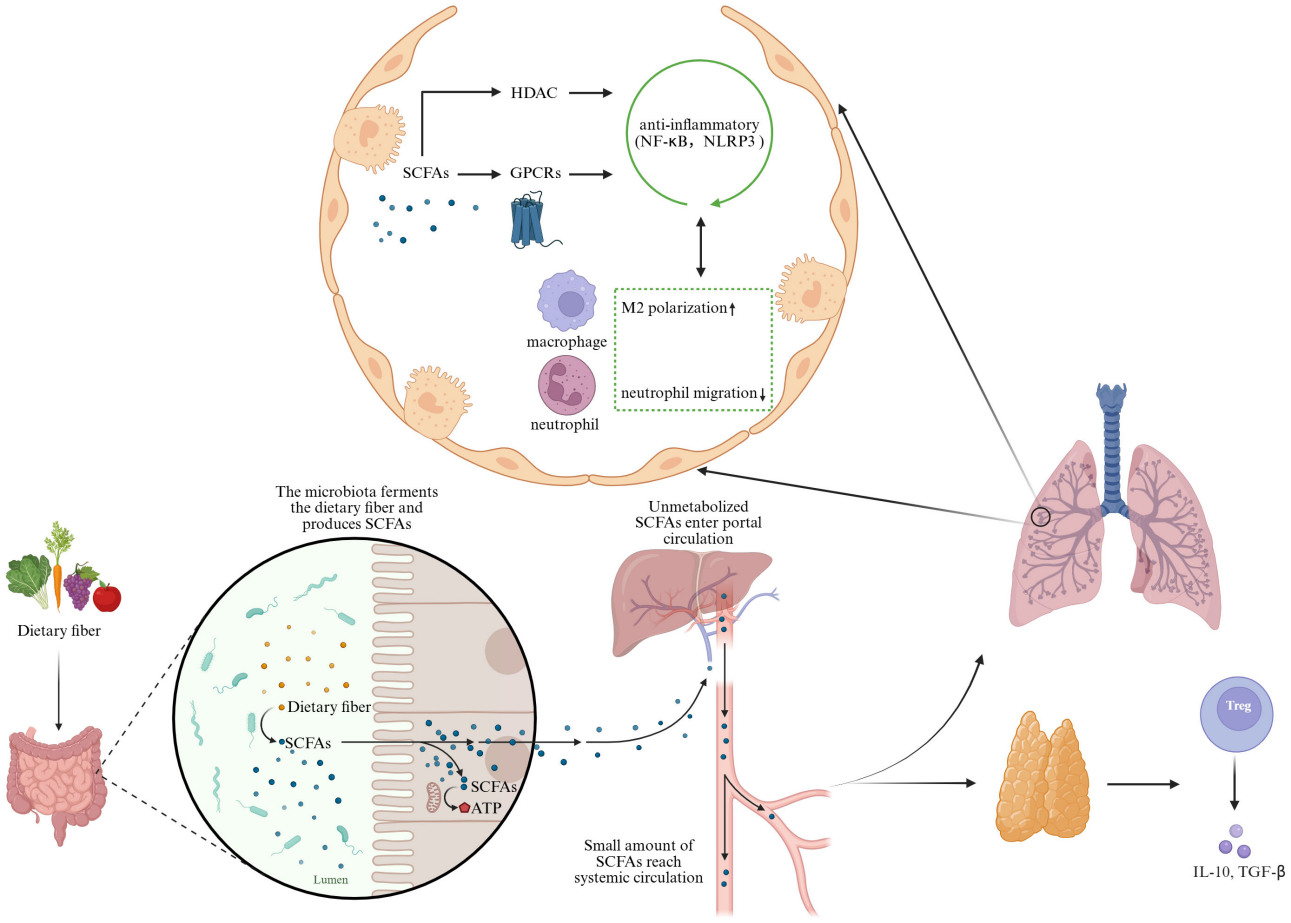
SCFAs entering the bloodstream can serve as potential therapeutic targets for preventing and treating ALI/ARDS by modulating systemic immune function and the innate immune function of the lung. ATP, adenosine triphosphate; GPCRs, G protein-coupled receptors; HDACs, histone deacetylases; IL, interleukin; NF-κB, nuclear factor kappa B; NLRP3, NOD-like receptor thermal protein domain associated protein 3; SCFAs, short-chain fatty acids; TGF, transforming growth factor; Treg, regulatory T cells.

SCFAs regulate pulmonary immune responses in ALI/ARDS through three core non-redundant molecular mechanisms: activating G protein-coupled receptors (GPCRs) - (GPR41, GPR43, GPR109a) to inhibit pro-inflammatory cytokine secretion (Ganapathy et al., 2013; Kim et al., 2013; Macia et al., 2015; He et al., 2020); regulating histone deacetylases (HDACs) activity for epigenetic suppression of pro-inflammatory gene transcription (Licciardi et al., 2011; Íñiguez-Gutiérrez et al., 2020); enhancing Treg cell differentiation by inhibiting c-Jun N-terminal kinase (JNK)1 and p38 pathways to regulate adaptive immunity (Haghikia et al., 2015) (**Figure 3**).

SCFAs exert hierarchical bidirectional regulation on almost all key immune cell circuits and structural cells in the lungs. In innate immune cells, SCFAs suppress monocytes/antigen-presenting cells (APCs) inflammatory responses and dendritic cells (DCs) maturation, reduce monocyte adhesion and migration, balance type 3 ILCs (ILC3s)/type 2 ILCs (ILC2s) functions, upregulate IL-22 production in both CD4^+^ T cells and ILCs, and regulate neutrophil apoptosis, neutrophil extracellular traps (NETs) formation and chemotaxis (Cox et al., 2009; Liu et al., 2012; Aguilar et al., 2014; Íñiguez-Gutiérrez et al., 2020; Suzuki et al., 2020; Yang et al., 2020; Kim, 2021; Li G. et al., 2021; Xiao et al., 2021; Eslick et al., 2022; Xuan et al., 2024). In adaptive immune cells, SCFAs regulate T/B cell differentiation: propionate reduces Th17/Th1 cells, acetate and butyrate promote Treg generation, and propionate drives B cell differentiation into plasma cells to enhance SIgA secretion (Atarashi et al., 2011; Atarashi et al., 2013; Smith et al., 2013; Wu et al., 2017; Martin-Gallausiaux et al., 2018; Haase et al., 2021; Lee et al., 2022). Moreover, SCFAs directly protect pulmonary endothelial and epithelial cells, inhibit DAMP release, reduce vascular leakage, and alleviate inflammatory lung injury (Moretti et al., 2009; Zhang et al., 2015; Li et al., 2018; Zhao et al., 2018; Karoor et al., 2021).

#### 3.2.2 BAs

Bile acids (BAs) are amphipathic cholesterol metabolites synthesized in hepatocytes via the classical [cholesterol 7α-hydroxylase (CYP7A1)-mediated] and alternative (sterol 27-hydroxylase (CYP27A1)-mediated) pathways (Collins et al., 2023). Primary BAs [cholic acid (CA) and chenodeoxycholic acid (CDCA) in humans] are reabsorbed via enterohepatic circulation, and the rest are metabolized into secondary BAs [deoxycholic acid (DCA) and lithocholic acid (LCA)] by gut bacteria in the colon (Hamilton et al., 2007). Gut microbiota dysbiosis in critically ill patients disrupts BAs metabolism and alters circulating BAs profiles (Kean et al., 2022).

Clinical studies confirm BAs dysregulation in ARDS patients (elevated primary BAs, reduced secondary BAs), and the serum glycine-taurine conjugation ratio correlates with patient prognosis (Harnisch et al., 2022; Harnisch et al., 2023). In the context of the gut-lung axis, BAs exert immunomodulatory effects mainly by binding to G protein-coupled bile acid receptor 1 (TGR5) expressed on pulmonary macrophages and DCs (Thibaut and Bindels, 2022). BA-mediated TGR5 activation suppresses LPS-induced NF-κB signaling (Wang et al., 2011), while accumulated primary BAs act as DAMPs to activate the NLRP3 inflammasome, trigger cytokine storms, damage alveolar epithelial cell mitochondria, and aggravate lung injury, reflecting their bidirectional regulatory role in ALI/ARDS (De Luca et al., 2022).

#### 3.2.3 Other key gut microbial metabolites

Besides SCFAs and BAs, multiple gut microbial metabolites supplement the gut-lung axis mechanistic framework in ALI/ARDS. Indole-related substances from tryptophan metabolism activate aryl hydrocarbon receptor (AhR) in lung macrophages and DCs to inhibit airway inflammation and promote immune tolerance (Gutiérrez-Vázquez and Quintana, 2018; Li L. et al., 2024); kynurenine restricts excessive T cell activation in the lungs (Chen et al., 2022; Renga et al., 2023). Elevated circulating succinate activates succinate receptor 1 (SUCNR1) on AMs to induce M1 polarization and mitochondrial damage (Wang Y. et al., 2023). Trimethylamine N-oxide (TMAO) activates the NLRP3 inflammasome and NF-κB pathway in AMs, triggers pyroptosis, and damages the pulmonary vascular endothelial barrier (Cao et al., 2024).

### 3.3 Critical immune cell circuits mediating gut-lung communication

The gut-lung axis signifies the bidirectional regulatory crosstalk between the intestinal and pulmonary immune systems; whereby gut microbial signals influence pulmonary innate and adaptive immune responses via a series of highly conserved immune cell circuits. These circuits further determine the onset, progression, and resolution of pneumonia- and sepsis-associated ALI/ARDS. These immune cell circuits are the downstream effectors of the two core pathways mentioned above, and their functional state directly determines the outcome of ALI/ARDS. In this section, we methodically analyze the key immune cell circuits that regulate gut-lung communication, providing in-depth mechanistic interpretations.

#### 3.3.1 ILCs: the core effector circuit of gut-lung immune crosstalk

ILCs are the core innate immune cells at mucosal surfaces, and their migratory properties, functional plasticity, and cytokine secretion capacity form the most direct and rapid effector circuit of gut-lung immune communication, which is the key link for gut microbiota to regulate pulmonary anti-infection immunity.

##### 3.3.1.1 ILC3s: the mucosal protective circuit of gut-lung axis

Gut commensal microbiota is the primary regulator of the ILC3 circuit. Intestinal mucosal DCs sense commensal bacterial signals and induce IL-22-secreting ILC3 migration to the lungs, establishing pulmonary mucosal immunity (Gray et al., 2017; Tamburini and Clemente, 2017). Segmented filamentous bacteria (Sfb) enhances anti-pneumonia immunity by expanding intestinal IL-22/IL-17A-secreting ILC3s (Gauguet et al., 2015), and SCFAs enhance ILC3 function via phosphatidyqinositolng kinase (PI3K)/protein kinase B (AKT) and mammalian target of rapamycin (mTOR) pathways (Kim, 2021). ILC3s exert dual pulmonary protection: IL-22 enhances epithelial barrier function and bacterial clearance, IL-17A promotes bacterial phagocytosis and resolves late inflammation (Van Maele et al., 2014; Xiong et al., 2016). Sepsis induces ILC1/ILC3 apoptosis to impair anti-infection capacity (Cruz-Zárate et al., 2018), while excessive IL-17A from ILC3s aggravates neutrophil infiltration and lung injury (Xie et al., 2024).

##### 3.3.1.2 ILC2s: the migratory regulatory circuit of gut-lung axis

ILC2s account for 30% of ILCs in adult human lungs and are the dominant subset in steady-state pulmonary tissue (Monticelli et al., 2011; De Grove et al., 2016). Their gut-to-lung trafficking depends on sphingosine-1-phosphate (S1P) signaling, and reduced plasma S1P in sepsis drives ILC2 migration to the lungs (Huang et al., 2018; Germain and Huang, 2019; Winkler et al., 2019); SCFAs inhibit ILC2 activity to balance pulmonary immunity (Kim, 2021). The ILC2 circuit plays a bidirectional role: IL-33-activated ILC2s upregulate IL-5 to aggravate injury, while secreting IL-9 to inhibit pyroptosis and alleviate ALI (Lai et al., 2018; Xu et al., 2018); ILC2 preactivation protects against ALI by inducing pulmonary eosinophilia (Krishack et al., 2019).

#### 3.3.2 AMs: the effector and long-term immune memory circuit

AMs are the most abundant innate immune cells in alveoli (90-95% of lung immune cells under homeostasis) and the primary recipients of gut-derived signals (Kopf et al., 2015). Their polarization and metabolic state directly determine ALI/ARDS progression (Wang and Wang, 2023). Gut microbiota depletion impairs AMs function, which is restorable by FMT (Schuijt et al., 2016). Gut microbiota regulates AMs via two pathways: gut-derived PAMPs directly activate pro-inflammatory programs, and metabolites regulate AM metabolic reprogramming and polarization (Wang Z. et al., 2020).

Metabolic reprogramming is the core mechanism: resting AMs rely on mitochondrial oxidative phosphorylation (OXPHOS) for energy homeostasis, while sepsis stabilizes HIF-1α to switch metabolism to glycolysis, driving M1 polarization and massive pro-inflammatory cytokine secretion (Kelly and O'Neill, 2015; Corcoran and O'Neill, 2016; Ben-Sahra and Manning, 2017; Zhang J. et al., 2024). SCFAs (especially butyrate) reverse this process, promote M2 polarization via PPAR-γ, HIF-2α, PI3K/Akt1 and TGF-β pathways, inhibit NF-κB and JAK2/STAT3 pathways, enhance anti-inflammatory cytokine secretion, and regulate AM-alveolar epithelial cell communication to resolve inflammation (Bissonnette et al., 2020; Chen et al., 2020; Wang Z. et al., 2020; Liu et al., 2021; MaChado et al., 2022; Panzer, 2022; Lun et al., 2024).

#### 3.3.3 Neutrophils

Neutrophils are the first recruited innate immune cells in injured lungs, and their functional state determines early inflammatory injury. Sepsis-induced intestinal barrier disruption causes gut bacterial translocation, leading to the accumulation of apoptosis-resistant neutrophils in the lungs, which obstruct microcirculation and aggravate ALI (Fialkow et al., 2006; Juss et al., 2016; Park et al., 2019). Gut dysbiosis expands pro-inflammatory Ly6G^+^CD11b^hi^ low-density neutrophils via endogenous LPS, enhancing NETs formation and endothelial damage (Takizawa et al., 2021). Gut-derived signals regulate neutrophils at multiple levels: acetate inhibits neutrophil apoptosis (Xuan et al., 2024), butyrate suppresses NETs formation (Íñiguez-Gutiérrez et al., 2020; Li G. et al., 2021), and gut LPS promotes neutrophil infiltration via TLR4/NF-κB signaling (Wang Y. et al., 2020; Li C. et al., 2024).

#### 3.3.4 DCs: the antigen presentation and signal transduction circuit of gut-lung axis

DCs act as core “signal translators”, linking gut microbial signals and pulmonary adaptive immunity via antigen migration and metabolite-mediated regulation. DCs sample gut microbial antigens and present them to pulmonary T cells (Maestroni, 2002; Kopf et al., 2015); gut metabolites (secondary BAs, tryptophan metabolites, SCFAs) inhibit DCs maturation, weaken antigen-presenting capacity, induce a tolerogenic phenotype, and regulate pulmonary adaptive immunity initiation (Liu et al., 2012; Gutiérrez-Vázquez and Quintana, 2018; Eslick et al., 2022; Thibaut and Bindels, 2022; Li L. et al., 2024).

#### 3.3.5 Adaptive immune cells

Gut microbial signals regulate pulmonary adaptive immunity mainly by balancing Th17/Treg cells. FMT and SCFAs restore Th17/Treg balance via the JAK/STAT pathway to reduce excessive immune activation (Haghikia et al., 2015; Zhang et al., 2025a). Innate-like adaptive subsets also participate: intestinal memory γδT17 cells migrate to the lungs and secrete IL-17A to aggravate inflammation (Xie et al., 2024); mucosal-associated invariant T (MAIT) cells are activated by riboflavin metabolites to enhance antibacterial immunity (Li et al., 2025a). SCFAs regulate B cells differentiation and SIgA secretion to strengthen mucosal humoral immunity (Wu et al., 2017; Martin-Gallausiaux et al., 2018).

### 3.4 Limitations of current mechanistic research

**Figure 4:**
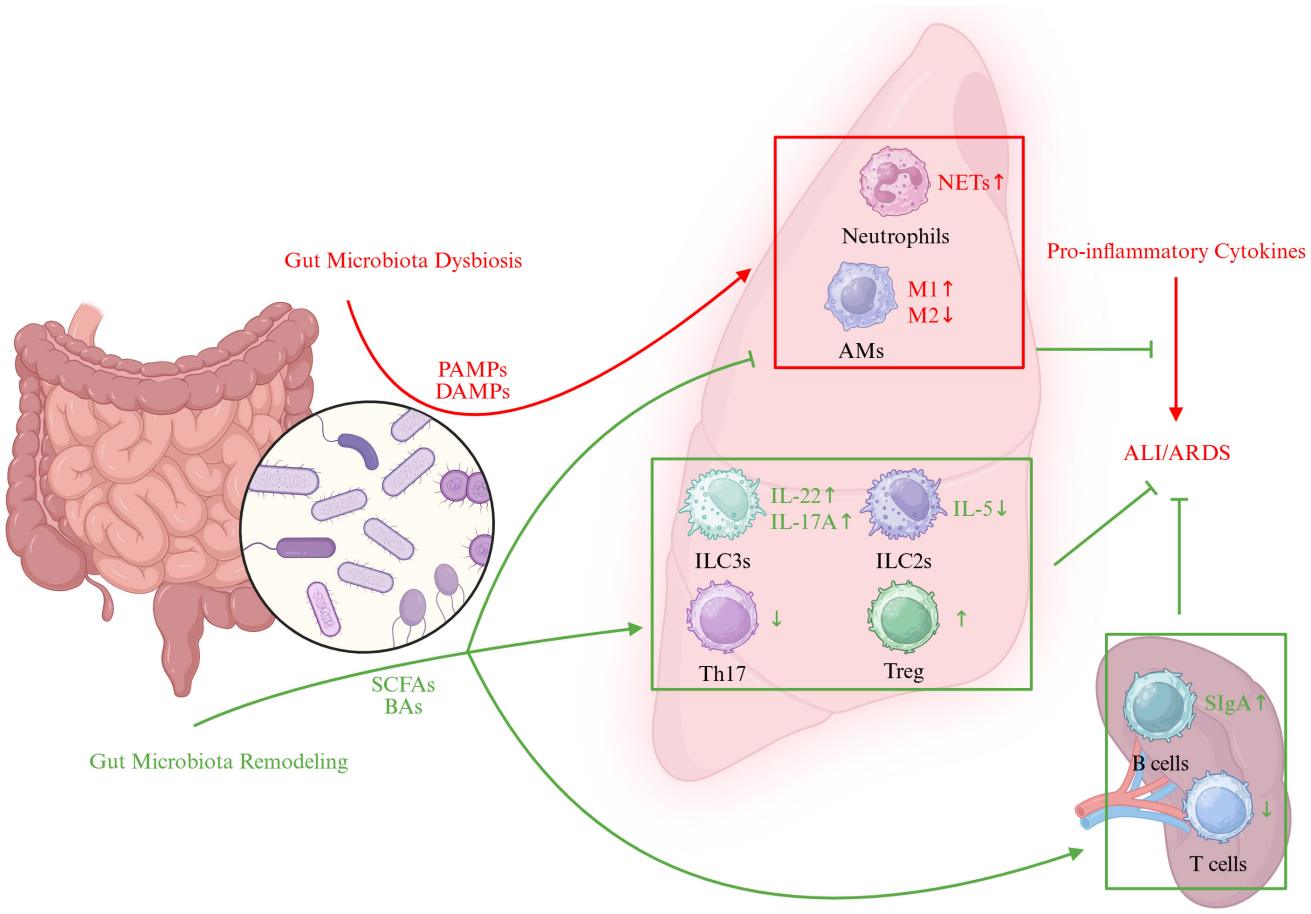
Gut microbiota modulates pulmonary innate and adaptive immune responses in ALI/ARDS along the gut-lung axis. ALI, acute lung injury; AMs, alveolar macrophages; ARDS, acute respiratory distress syndrome; BAs, bile acids; DAMPs, damage-associated molecular patterns; IL, interleukin; ILCs, innate lymphoid cells; ILC2s, type 2 ILCs; ILC3s, type 3 ILCs; NETs, neutrophil extracellular traps; PAMPs, pathogen-associated molecular patterns; SCFAs, short-chain fatty acids; SIgA, secretory IgA; Th17, T helper cell 17; Treg, regulatory T cells. ↑ mean increase or activate, and ↓ mean decrease or inhibit.

The aforementioned evidence confirms that gut microbiota modulates pulmonary innate and adaptive immune responses in ALI/ARDS via well-defined cellular and molecular immune circuits along the gut-lung axis (**Figure 4**). These mechanistic findings provide a clear theoretical basis for microbiota-targeted therapeutic strategies for ALI/ARDS.

Despite the well-established mechanistic framework of the gut-lung axis in ALI/ARDS, there are still key limitations in current research that restrict the translation of basic findings into clinical applications. First, given the existence of marked species differences, the clinical translatability of mechanistic findings is limited. This is due to the fact that the majority of current mechanistic findings are derived from *in vitro* experiments and mouse models. Significant species differences exist in gut microbiota composition and immune system function between mice and humans. Consequently, there is considerable uncertainty regarding the translatability of these mechanisms to clinical ARDS patients. Second, the majority of studies have placed excessive emphasis on single pathways and have neglected to research complex microbial network interactions. The majority of studies have focused on a single microbial metabolite or signaling pathway. The synergistic or antagonistic effects of complex gut microbial signals (multiple components, multiple metabolites, multiple pathways) in the gut-lung axis remain poorly characterized. In particular, the intricate network regulatory effects of SCFAs combined with other functional metabolites have not been fully elucidated (Wang et al., 2022). Thirdly, the relative contribution of the two core pathways (direct PAMPs activation vs. DAMPs activation) in different etiologies of ARDS (sepsis, trauma, viral infection, etc.) has not been clearly defined. This limitation restricts the development of etiology-specific targeted therapies. Fourthly, the heterogeneity of the individual subjects limits the precision of intervention due to the highly individual effects of targeted microbiota modulation. These effects are caused by the heterogeneity of gut microbiota composition among different individuals and the complex interaction between gut bacteria and the host. This, in turn, increases the difficulty in translating basic research findings into clinical treatment strategies for ARDS.

## 4 Microbiota-targeted therapeutic strategies for ALI/ARDS

In accordance with the mechanistic framework of the gut-lung axis in ALI/ARDS, a substantial body of preclinical research has validated the efficacy of microbiota-targeted interventions in mitigating ALI/ARDS. These interventions have been shown to restore gut microbiota homeostasis, repair the intestinal barrier, and regulate the pulmonary immune response, thereby promoting a state of homeostasis in the gut microbiota. In this section, we systematically summarize the latest research progress of microbiota-targeted therapeutic strategies, including probiotics, prebiotics, FMT, and TCHMs.

### 4.1 Core rationale of microbiota-targeted interventions

As previously mentioned, gut microbiota dysbiosis is a causal driver of ALI/ARDS progression via the gut-lung axis. The primary objectives of microbiota-targeted interventions are consistent with the pathological mechanisms of ALI/ARDS. These objectives are as follows: (1)Restore the eubiosis of the gut microbiota to reverse dysbiosis-associated pathological changes; (2)Repair impaired intestinal barrier function to reduce bacterial translocation and systemic PAMP exposure; (3)Enhance the production of beneficial microbial metabolites (SCFAs, secondary BAs) to amplify anti-inflammatory signaling in the lungs; and (4)Modulate systemic and pulmonary immune responses via the gut-lung axis to mitigate excessive inflammatory injury in ALI/ARDS.

### 4.2 Probiotics, prebiotics, FMT, and TCHMs

#### 4.2.1 Probiotics

Probiotics are defined as live microorganisms that confer health benefits to the host by regulating gut microbiota composition and function. Preclinical studies have validated the protective effects of multiple probiotic strains against ALI/ARDS of various etiologies (**Table 4**) (Han et al., 2023; Shen et al., 2023b; Shen et al., 2023a; Xiong et al., 2023; Li et al., 2025b; Staley et al., 2025; Yao et al., 2025; Li et al., 2026). The following strains have been shown to have validated efficacy: *Akkermansia muciniphila* (Han et al., 2023; Xiong et al., 2023), *Lactobacillus reuteri* (Shen et al., 2023a), *Weissella cibaria* (Li et al., 2025b), *Bacteroides acidifaciens* (Staley et al., 2025), and SCFA (butyrate)-producing strains, including *Akkermansia muciniphila* (Shen et al., 2023b), and a mixture of probiotics (*Ligilactobacillus salivarius* H3, etc.) (Li et al., 2026). The following therapeutic mechanisms have been identified: Downregulation of Bacteroidetes and upregulation of Firmicutes at the phylum level (Shen et al., 2023b; Xiong et al., 2023), consistent with the dysbiosis pattern observed in ALI/ARDS (Li S. et al., 2021). Promotion of the production of SCFAs (butyric acid) to activate anti-inflammatory signaling in the lungs (Shen et al., 2023b; Li et al., 2026). Inhibition of NF-κB pathways (Shen et al., 2023b; Yao et al., 2025) to reduce pulmonary pro-inflammatory cytokine secretion. Modulation of the phenotype and function of pulmonary macrophages (Yao et al., 2025; Li et al., 2026) and neutrophils (Shen et al., 2023b; Shen et al., 2023a). The suppression of excessive inflammatory infiltration is achieved through the following mechanisms: upregulation of intestinal barrier proteins (ZO-1, occludin, MUC2) to protect gut barrier function (Shen et al., 2023b; Shen et al., 2023a; Li et al., 2025b; Yao et al., 2025).

**Table 4:** Probiotics used in the treatment of ALI/ARDS.

Names	Induction method of models	Remodeling effect of the most relevant gut microbiota	Remodeling effect of the most relevant lung microbiota	Relevant signaling molecules/pathways of intestinal barrier	Gut microbiota-derived metabolites	Relevant signaling molecules/pathways of inflammatory response	Ref.
Live *Akkermansia muciniphila* and pasteurized *Akkermansia muciniphila*.	C57BL/6 mice. 50 μL LPS (1 mg/mL) i.t.	Bacteroidetes↓, Firmicutes↑, *Allobaculum*↑, *Bifidobacterium*↑, *Clostridium*↑, *Lactobacillus*↑.	Not listed.	Not listed.	Not listed.	Not listed.	(Xiong et al., 2023)
Amuc_1100 protein from *Akkermansia muciniphila*.	SD rats. CLP.	Observed species index↑, Chao1 index↑. Tenericutes↓, Verrucomicrobia↑, *Akkermansia*↑, *Bacteroides*↓, *Escherichia*-*Shigella*↓, *Lactobacillus*↑, *Parabacteroides*↓, *Romboutsia*↑.	Not listed.	Not listed.	Not listed.	Not listed.	(Han et al., 2023)
*Lactobacillus reuteri*.	C57BL/6 mice. 0.5 mL LPS (5 mg/kg) i.t.	Verrucomicrobiota↑, Verrucomicrobiae↑, Verrucomicrobiales↑, Akkermansiaceae↑, *Citrobacter*↓.	Not listed.	Colon: ZO-1↑, occludin↑, MUC2↑. Serum: LBP↓.	Not listed.	Lung: macrophages↓, neutrophils↓.	(Shen et al., 2023a)
*Akkermansia muciniphila*.	C57BL/6 mice. LPS (5 mg/kg) i.t.	The ratio of Firmicutes/Bacteroidotas↑. Fusobacteriata↓, *Akkermansia*↑, ASF356↑, *Bacteroides*↓, *Helicobacter*↑, *Klebsiella*↓, *[Eubacterium] coprostanoligenes* group↓, *Roseburia*↑, *Ruminococcus*↓.	*Dubosiella*↑, *Lactobacillus*↑, *Methylobacterium–Methylorubrum*↓, *Muribacter*↓.	Colon: ZO-1↑, occludin↑. Serum: LBP↓.	Butyric acid↑.	Lung: macrophages↓, neutrophils↓. TLR2/Myd88/NF-κB pathway↓.	(Shen et al., 2023b)
Mixed probiotics combination (*Ligilactobacillus salivarius* H3, *Bacillus stratosphericus* J1366, *Priestia megaterium* J1037).	C57BL/6 J mice. MRSA suspension (50 µL, 1 × 10^9^ CFU/mL) i.n.	*Bifidobacterium*↑, *Clostridium* sensu stricto 1↓, *Candidatus arthromitus*↓, *Dubosiella*↑, *Escherichia-Shigella*↓, Lachnospiraceae NK4A136↓, *Lactobacillus*↑, *Ligilactobacillus*↑, *Staphylococcus*↓.	Not listed.	Not listed.	Butyric acid↑.	Lung: macrophage (M1↓, M2↑).	(Li et al., 2026)
Pasteurized *Weissella cibaria*.	Mice. CLP.	Bacteroidota↑, Campylobacterota↓, Firmicutes↑, Muribaculaceae↑, Proteobacteria↓, *Escherichia-Shigella*↓, *Helicobacter*↓.	Not listed.	Colon: apoptosis↓, occludin↑, Claudin-1↑.	Not listed.	BALF: leukocytes↓, neutrophils↓.	(Li et al., 2025b)
*Escherichia coli* Nissle 1917.	Balb/c mice. LPS (5 mg/kg) i.v.	The caecum was the predominant site of bacterial colonization.	Not listed.	Colon: ZO-1↑.	Not listed.	MLNs: CD8^+^ T (Tc)17 cells↓. Lung: macrophage (M1↓, M2↑), neutrophils↓, TLR4/NF-κB pathway. Blood: neutrophils↓.	(Yao et al., 2025)
*Bacteroides acidifaciens*.	C3H/HeJ mice. 5 μg SEB i.t., 2 hours later 2 μg SEB i.p.	*Bacteroides acidifaciens*, a common gut commensal.	Not listed.	BA enhance gut barrier function in males but impairing it in females.	Not listed.	BA promote anti-inflammatory pathways in males and inflammatory skewing in females.	(Staley et al., 2025)

↑ mean increase, and ↓ mean decrease.

The clinical translation of probiotic therapies has yielded equivocal results. A multicenter randomized controlled trial (RCT, NCT030745552) found that *B. subtilis* and *E. faecalis* are effective and safe for preventing ventilator-associated pneumonia (VAP) in critically ill patients (Zeng et al., 2016), while another RCT (NCT02462590) found no significant preventive effect of *Lactobacillus rhamnosus* GG on VAP incidence (Johnstone et al., 2021). This heterogeneity is likely driven by differences in probiotic strains, intervention timing, baseline disease severity, and pre-existing gut microbiota composition of enrolled patients. Relevant clinical trials (NCT06092554) investigating probiotic interventions for VAP are currently ongoing (Corriero et al., 2025). The negative outcome of the NCT02462590 trial may be ascribed to three primary factors: the single *Lactobacillus rhamnosus* GG strain exhibited low colonization efficiency in critically ill patients receiving broad-spectrum antibiotics; the intervention was initiated after critical illness onset, at which point intestinal barrier damage had already been established; and the trial did not stratify patients by baseline gut microbiota composition, which directly affects probiotic efficacy. Notably, the use of probiotics in critically ill patients has been associated with potential safety risks. Multiple studies have reported that probiotic use in immunosuppressed or intestinal barrier-impaired patients may result in probiotic bacteremia and even sepsis, particularly in cases of *Lactobacillus* GG (Vahabnezhad et al., 2013; Koyama et al., 2019; Kullar et al., 2023). A retrospective cohort study found that probiotic use in ICU patients with central venous catheters is associated with increased mortality, especially for powder formulations (Mayer et al., 2023). Consequently, the clinical application of probiotics in ALI/ARDS patients necessitates rigorous indication control, and large-scale RCTs are imperative to substantiate its efficacy and safety.

#### 4.2.2 Prebiotics

Prebiotics are non-digestible food ingredients that beneficially affect the host by selectively stimulating the growth and/or activity of one or a limited number of bacteria in the colon, thus improving host health. The most commonly studied prebiotics include inulin, fructooligosaccharides (FOS), and resistant starch, which are the main substrates for gut bacteria to produce SCFAs.

Preclinical studies have demonstrated the efficacy of prebiotic supplementation in alleviating systemic inflammation. FOS has been demonstrated to enhance the abundance of Bifidobacterium within the gastrointestinal tract, thereby fortifying the intestinal barrier and attenuating systemic inflammation (Cani et al., 2009). The addition of long-chain inulin and resistant starch to a subject’s diet has been demonstrated to have a positive effect on the levels of SCFA-producing bacteria in the gastrointestinal tract (Canfora et al., 2022). This, in turn, has been shown to increase the levels of SCFAs in the bloodstream (Canfora et al., 2022). However, the clinical translation of prebiotics is encumbered by significant challenges. A phase II RCT in ICU sepsis patients demonstrated that a single inulin intervention was safe but had no substantial clinical benefit (Park et al., 2025). The primary reasons for the inconsistent efficacy include the significant individual heterogeneity of the baseline gut microbiota in critically ill patients and the inability of single prebiotics to meet the needs of different patients. Future clinical application of prebiotics requires personalized formulation based on the patient’s gut microbiota characteristics.

#### 4.2.3 FMT

FMT is defined as the transfer of fecal microbiota from a healthy donor into the gastrointestinal tract of a recipient, with the objective of restoring gut microbiota homeostasis. Preclinical studies have confirmed that FMT can effectively alleviate ALI/ARDS in multiple animal models. The mechanisms by which FMT exerts its therapeutic effects include the restoration of the gut microbiota-SCFAs axis, the enhancement of the bactericidal ability of AMs, the inhibition of the TLR4/NF-κB inflammatory pathway, and the restoration of the Th17/Treg balance (Wen et al., 2022; Tang et al., 2024; Oliwa-Libumska et al., 2025). As demonstrated by clinical case reports, FMT has been shown to effectively treat refractory multidrug-resistant bacterial infections in critically ill patients and reduce the risk of secondary pulmonary infections (Oliwa-Libumska et al., 2025).

However, the clinical application of FMT in ALI/ARDS remains in the exploratory stage, encountering numerous challenges: (1)The absence of standardized donor selection criteria, fecal preparation protocols, and administration methods; (2)The potential risk of iatrogenic infection, especially in immunosuppressed critically ill patients; (3)The significant individual heterogeneity of colonization efficacy; (4)The lack of large-scale RCTs to verify its efficacy and safety in ALI/ARDS patients. Consequently, the use of FMT as a conventional treatment for ALI/ARDS is not currently recommended. Its utilization is restricted to cases that are refractory to other treatment options and is permitted only under strict ethical approval and standardized operational procedures.

#### 4.2.4 TCHMs

The efficacy of TCHMs in mitigating ALI/ARDS is well-documented, with a mechanistic framework analogous to that of probiotics, prebiotics, and FMT. In all three of these interventions, the modulation of gut microbiota serves as the fundamental initiating factor. The multi-level protective effects of TCHMs in ALI/ARDS are achieved through three interrelated pathways targeting the gut microbiota and its downstream immune cascades (**Table 5**) (Alghetaa et al., 2021; Song et al., 2021; Huo et al., 2022; Li et al., 2022; Tang et al., 2022; Bao et al., 2023; Ding et al., 2023; Hu T. et al., 2023; Li et al., 2023; Qiu et al., 2023; Wang Z. et al., 2023; Xiong et al., 2023; Cao et al., 2024; Chen et al., 2024; Guo K. et al., 2024; He et al., 2024; Hu et al., 2024; Li R. et al., 2024; Li Z. et al., 2024; Lu et al., 2024; Luo et al., 2024; Ma et al., 2024; Su et al., 2024; Xiao et al., 2024; Zhang L. et al., 2024; Cheng et al., 2025; Guo et al., 2025; Lu et al., 2025; Luo et al., 2025; Ni et al., 2025; Wang X. et al., 2025; Yang et al., 2025; Yin et al., 2025; Yu et al., 2025; Zeng et al., 2025; Zhong et al., 2025; Zhu et al., 2025).

**Table 5:** Traditional Chinese herbal medicines used in the treatment of ALI/ARDS.

Names	Induction method of models	Remodeling effect of the most relevant gut microbiota	Remodeling effect of the most relevant lung microbiota	Relevant signaling molecules/pathways of intestinal barrier	Gut microbiota-derived metabolites	Relevant signaling molecules/pathways of inflammatory response	Ref.
RES.	C3H/HeJ mice. SEB (5 μg/mouse) i.t. After 120 min SEB (2 μg/mouse) i.p.	Tenericutes↑, *Lactobacillus* spp.↑, *Lactobacillus reuteri↑.*	Actinobacteria↑, *Lactobacillus reuteri*↑.	Not listed.	SCFAs: propionic acid↑, iso-butyric acid↑, butyric acid↑	Lung: CTL cell↓, NKT cell↓. Spleen: Tregs↑, Th3 cell↑.	(Alghetaa et al., 2021)
Sinomenine.	ICR mice. CLP.	Chao index↑. Prevotellaceae UCG-001↑, *Escherichia-Shigella*↓.	Not listed.	Colon: occludin↑, ZO-1↑.	Not listed.	Colon: AhR/Nrf2 pathway↑.	(Song et al., 2021)
Rhubarb.	C57BL/6 mice. LPS (3 mg/kg).	*Alistipes*↑*, Bacteroides*↓, *Clostridium*↑, *Intestinimonas*↑, *Lactobacillus*↑.	Not listed.	Not listed.	SCFAs (not tested).	Serum: HDAC6↑. Lung: HDAC6↑. Spleen: Th17 cell↑, Treg cell↓.	(Tang et al., 2022)
GQD.	SD rats. LPS (5 mg/kg) i.t.	Firmicutes↑, Ruminococcaceae UCG-007↑, *Bacteroides*↓.	Not listed.	Not listed.	SCFAs: acetic acid↑, propionic acid↑, butyric acid↑.	Lung: C3↓, C5a↓, IL-17↓, TGF-β↑, CYP1A1↑.	(Li et al., 2022)
XBCQ.	BALB/c mice. H1N1 at the dose of 2LD50 (suspended in 30 μL of the RPMI-1640 medium) i.n.	Firmicutes↑, Verrucomicrobia↓, Akkermansiaceae↓, Enterobacteriaceae↓, Lachnospiraceae↑, Ruminococcaceae↓, f_Lachnospiraceae_Unclassified↑, *Anaerostipes*↑, *Akkermansia*↓, *Blautia*↑, *Cuneatibacter*↓, *Escherichia-Shigella*↑, *Erysipelatoclostridium*↑, *Flavonifractor*↓, *Proteus*↓.	Not listed.	Colon: intestinal injury↓.	SCFAs (not tested).	Lung: TLR7/MyD88/NF-κB pathway↓.	(Huo et al., 2022)
KQ.	C57BL/6 mice. 50 μL LPS (1 mg/mL) i.t.	Bacteroidetes↓, Firmicutes↑, *Akkermansia muciniphila*↑.	Not listed.	Not listed.	SCFAs: acetate↑, propionate↑.	Not listed.	(Xiong et al., 2023)
WRS.	C57BL/6 mice, 25 μL of SEB was administered intranasally by micropipette at a dose of 5 μg per mouse.	Deferribacteres↓, Tenericutes↓, Verrucomicrobia↑, Desulfovibrionaceae↑, Muribaculaceae↑, Rikenellaceae↓, Ruminococcaceae↑, *Akkermansia*↑, *Desulfovibrio*↓, *Helicobacter*↓.	Not listed.	Not listed.	SCFAs: acetic acid↑, propionic acid↑, butyric acid↑, valeric acid↑.	Lung: FFAR3↑, FFAR2↑.	(Hu T. et al., 2023)
Total ginsenosides from Panax ginseng stems.	C57BL/6J mice. LPS (5 mg/kg,100 μL/per mouse) i.t.	ACE index↑, Shannon index↑. Bacteroidetes↓, Firmicutes↑, Lachnospiraceae↑, Muribaculaceae↑, Prevotellaceae↓.	Not listed.	Not listed.	SCFAs: acetic acid↑, propionic acid↑, butyric acid↑, total SCFAs↑.	Not listed.	(Ding et al., 2023)
Baicalin.	SD rats. MDR *P. aeruginosa* (1 × 10^8^ CFU diluted in 200 µL saline) i.t.	Chao1 index↑, Shannon index↑. Muribaculaceae↓, *Alistipes*↓, *Bacteroides*↑, *Lactobacillus*↑, *Ligilactobacillus*↑.	Not listed.	Not listed.	SCFAs (not tested).	Lung: TLR4/NF-κB pathway↓.	(Li et al., 2023)
JHD.	C57BL/6 mice. CLP.	Bacteroidetes↓, Desulfobacterota↓, Firmicutes↑, Christensenellaceae↓, Clostridiaceae↓, *Citrobacter*↓, *Enterococcus*↓, *Enterococcus faecalis*↓, *Escherichia coli*↓, *Escherichia-Shigella*↓.	*Escherichia coli*↓.	Colon: ZO-1↑, Muc-1↑.	Not listed.	BALF: neutrophils↓, γδT cells↓.	(Bao et al., 2023)
miR-7972 derived from fresh *R. Radix*.	BALB/c mice. LPS (32 mg/kg) i.t.	*Allobaculum*↓, *Escherichia-Shigella*↓, *Prevotella*↑.	Not listed.	Not listed.	Not listed.	Lung: GPR161↓. BALF: macrophage (M1↓, M2↑).	(Qiu et al., 2023)
QYD.	C57BL/6 mice. Caerulein (100μg/kg) i.p. 10 consecutive times every hour, LPS (10 mg/kg) i.p. with last injection.	*Akkermansia*↑, *Bacteroides*↑, *Enterobacter*↓, *Enterococcus*↓, *Escherichia*↓, *Helicobacter*↓, *Parabacteroides*↑, *Peptostreptococcus*↓, *Prevotella*↑, *Roseburia*↑.	Not listed.	Ileum: ZO-1↑, occludin↑. Serum: D-LAC↓, LPS↓, DAO↓.	Plasma and lung: SCFAs (propionate↑, butyrate↑, acetate↓).	AMPK↑/NF-κB↓/NLRP3↓ pathway.	(Wang Z. et al., 2023)
FZJDF.	BALB/c mice. LPS (0.5 mg/kg) i.n.	Invsimpson index↓. Bacteroidales↓, *Candidatus Amulumruptor*↓, *Parabacteroides*↓.	Not listed.	Not listed.	Not listed.	Lung: macrophage (M1↓)	(Lu et al., 2024)
HEL.	SD rats. 3 mg/kg LPS (40 mg of LPS dissolved in 20 mL normal saline and the injection volume as 1.5 mL/kg) i.t.	Bacteroidota↑, Desulfobacterota↓, Firmicutes↓, Patescibacteria↓, Proteobacteria↑, Spirochaetota↓, *Clostridium sensu stricto 1*↓, *Lactobacillus*↓, *Romboutsia*↓, *Treponema*↓.	Not listed.	Not listed.	Not listed.	Lung: TLR4/NF-κB/NLRP3 pathway↓.	(Li R. et al., 2024)
YZC.	Balb/c mice. LPS (30 mg/kg) i.p.	Shannon index↑, Simpson index↑. Firmicutes/Bacteroidetes ratio↓. Bacteroidetes↑, Proteobacteria↓, Prevotellaceae UCG-001↑, *Akkermansia*↑, *Alloprevotella*↓, *Cetobacterium*↓, *Kurthia*↓, *Methylobacterium*↓, *Parabacteroides*↑.	Not listed.	Colon: ZO-1↑.	Serum and lung: TMAO↓.	Lung: NF-κB/NLRP3 pathway↓.	(Cao et al., 2024)
JFG.	C57BL/6J mice. BLM (3 mg/kg) i.t.	Shannon index↑. Firmicutes/Bacteroidetes ratio↓. Lachnospiraceae NK4A136_group↑, *Akkermansia*↑.	Not listed.	Not listed.	SCFAs (not tested).	Not listed.	(Xiao et al., 2024)
BBP.	C57BL/6 mice. 20 µL of *Staphylococcus aureus* solution (bacterial concentration of 1 × 10^−7^ CFU/mL) i.n., 100 µL of LPS solution (1 mg/mL) i.t.	Firmicutes/Bacteroidetes ratio↓. Bacteroidetes↑, Deferribacteres↓, Firmicutes↓, Proteobacteria↓, uncultured bacterium f_Muribaculaceae↑, *Escherichia-Shigella*↓.	Not listed.	Not listed.	SCFAs: acetate↑, propionate↑, butyrate↑.	Lung: CD14/NF-κB pathway↓.	(Cheng et al., 2025)
EEP, EEG.	ICR mice. Heat-inactivated MRSA (20 mg/kg) i.v.	g_norank_f_Muribaculaceae↑, g_norank_f_norank_o_Clostridia_UCG-014↑, g_unclassified_f_Lachnospiraceae↑, *Akkermansia*↑, *Lactobacillus*↓, *Staphylococcus*↓.	Not listed.	Not listed.	SCFAs: acetic acid↑, butyric acid↑, propionic acid↑, total SCFAs↑.	Lung: macrophage↓, NF-κB pathway↓.	(Li Z. et al., 2024)
NDC.	Wistar rats. CLP.	Chao1 index↑, Shannon index↑, Simpson index↑. Firmicutes/Bacteroidetes ratio↓. Actinobacteria↓, Gammaproteobacteria↓, Enterobacteriaceae↓, Lachnospiraceae↑, *Ruminococcus*↑.	Not listed.	Not listed.	SCFAs (not tested).	GRP18↑. Lung: NF-κB pathway↓, NLRP3 pathway↓.	(He et al., 2024)
AS-IV.	SD rats. LPS dissolved in normal saline (5mg/kg, 100 μL/rat) i.t.	Chao1 index↑. L-AS-IV: Bacteroidetes↑, Firmicutes↓; M-AS-IV and H-AS-IV: *Lactobacillus↑*, *Prevotella↑*, *Roseburia↑.*	Not listed.	Colon: injury↓.	BAs (not tested).	Lung: PI3K/AKT/mTOR pathway↓.	(Luo et al., 2024)
FZQS.	C57BL/6 mice. 30 μL of LPS (1 mg/mL) i.n.	norank_f_Muribaculaceae↑, unclassified_f_Lachnospiraceae↑, uncultured_f_Lachnospiraceae↑, *Blautia*↓, *Corynebacterium*↓, *Klebsiella*↑, *Lactobacillus*↑, *Psychrobacter*↓, *Staphylococcus*↓.	Not li.sted.	Not listed.	Not listed.	Peripheral blood: macrophage (M1↓, M2*↑*), activated neutrophils↓. Lung: NF-κB↓, p–NF–κB↓, RANKL↓, CXCL1↓, MADCAM1↓, CCL19↓, CCR8↓, ICAM-1↓.	(Guo K. et al., 2024)
HupA.	C57BL/6 mice, and *α7nAChR*^-^*^/^*^-^ mice. CLP.	*Akkermansia*↑, *Escherichia*↓.	Not listed.	Colon: injury↓.	SCFAs: acetic acid↑, propionic acid↑.	Lung: α7nAChR↑, NF-κB pathway↓.	(Su et al., 2024)
DYY.	SD rats. LPS (10 mg/kg) i.p.	Simpson index↑. Bacteroidota↓, Desulfobacterota↑, Firmicutes↑, Patescibacteria↓, uncultured Lachnospiraceae↓, *[Eubacterium] xylanophilum_group*↓, *Enterorhabdus*↓, *Lactobacillus*↑, *NK4A214_group*↓, *Romboutsia*c, *Ruminococcus*↓, *Turicibacter*↑, *UCG-005*↑.	Not listed.	Not listed.	SCFAs (not tested).	Not listed.	(Zhang L. et al., 2024)
BAI.	C57BL/6 mice. 25 μl of SEB at a concentration of 0.2 μg/μl i.t., 2 hours later SEB dose at a concentration of 0.02 μg/μl i.p.	BAI 30: Desulfovibrionaceae↑, Prevotellaceae_NK3B31↑. BAI 60: Bacteroidetes↑, Firmicutes↓, Proteobacteria↓, Clostridia↓, Lachnospiraceae↓, Muribaculaceae↑, Prevotellaceae↑, Lachnospiraceae_NK4A136↓, *Clostridia*↓, *Desulfovibrio*↓.	Not listed.	Not listed.	SCFAs: acetic acid↑, propionic acid↑, butyric acid↑, valeric acid↑.	Blood: CD4+ T cells↓, CD8+ T cells↓. Lung: FFAR2↑, FFAR3↑, TLR4/ MyD88 pathway↓.	(Hu et al., 2024)
RRFJ.	BALB/C mice, LPS (2 mg/kg) i.t.	Chao1 index↑, Shannon index↑, observed OTUs index↑. Bacteroidota↓, Proteobacteria↓, unidentified_Enterobacteriaceae↓, *Akkermansia*↑, *Bacteroides*↓, *Klebsiella*↓, *Ligilactobacillus*↑, *Limosilactobacillus*↑.	Not listed.	Intestine: injury↓; IL-6↓, IL-1β↓, TNF-α↓.	SCFAs: acetic acid↑, propionic acid↑, butyric acid↑.	Not listed.	(Chen et al., 2024)
TFXF.	Wistar rats. LPS (7.5 g/kg) i.p.	Observed OTUs index↑, Sobs index↑, Shannon index↑, Firmicutes↑, Proteobacteria↓, norank_f_Eubacterium_coprostanoligenes_group↑, *Bacteroides*↓, *Ruminococcus*↑.	Not listed.	Colon: injury↓; IL-1β↓, TNF-α↓.	SCFAs (not tested).	Lung: p38 MAPK/MLCK pathway↓.	(Ma et al., 2024)
CMME.	Balb/c mice. LPS (3 mg/kg) i.n.	Firmicutes/Bacteroidetes ratio↓. Actinobacteria↑, Desulfobacterota↓, unclassified_Lachnospiraceae↑, *Acinetobacter*↑, *Alloprevotella*↑, *Bacteroides*↓, *Odoribacter*↑, *Parabacteroides*↓.	Not listed.	Blood: WBC↓, LYM↓, NEU↓.	BAs: taurocholic acid↑, cholic acid↑, deoxycholic acid↑, chenodeoxycholic acid↑.	Lung: macrophage↓, neutrophils↓.	(Wang X. et al., 2025)
XFBD.	C57BL/6 mice. CLP.	Richness index↑, Chao1 index↑. Prevotellaceae↑, Ruminococcaceae UCG-014↑, *Dubosiella newyorkensis*↓, *Ileibacterium*↓, *Ileibacterium valens*↓.	Not listed.	Blood: LPS↓.	Not listed.	GPR18↑. Spleen: macrophage (M1↓). Lung: C/EBP-δ↓, NF-κB↓, NLRP3↓.	(Yang et al., 2025)
EBEE.	C57BL/6J mice. LPS (1µg/g of body weight) i.n.	Chthoniobacteraceae↑, Staphylococcaceae↓,	Not listed.	Not listed.	Not listed.	Lung: AGER↓, ITGB2↑.	(Yin et al., 2025)
BYTQ.	C57/B6 mice. LPS (5 mg/kg) i.t.	Ace index↑, Chao index↑, Sobs index↑. Bacteroidota↓, Bacillota↑, Lactobacillales↑, Prevotellaceae↓, *Dubosiella*↓, *Ligilactobacillus*↓.	Not listed.	Colon: injury↓; ZO-1↑, occludin↑. Serum: LPS↓, D-LA↓, DAO↓.	Not listed.	Lung: PI3K/AKT/mTOR pathway↑.	(Ni et al., 2025)
HCPM (*Houttuynia* pectin).	BALB/c mice. 30 μL 2 × LD_50_ H1N1 virus dilution i.n.	Simpson index, Shannon index↑, Pielou-e index↑. Bacteroidetes↑, Clostridiaceae bacterium↓, Lachnospiraceae bacterium↓, *Akkermansia muciniphila*↑, *Bacteroides caccae*↑, *Bacteroides eggerthii*↑, *Dorea* sp. ↓, *Eubacterium* sp. ↓, *Oscillibacter* sp. ↓, *P. vulgatus*↑.	Not listed.	Intestine: injury↓; TNF-α↓, IL-6↓, IFN-γ↓, IL-17 A↓, IL-10↑; MUC-2↑, ZO-1↑, occludin↑.	SCFAs: acetic acid↑, propionic acid↑, butyric acid↑.	Lung: Th17 CCR6/Treg CCR6↓, GPR43/JAK2/STAT3 pathway↓.	(Zhu et al., 2025)
LRP.	C57BL/6J mice. LPS (5 mg/kg) i.t.	Chao1 index↑, Pielou-J index↑, Shannon index↑, Simpson index↑. Bacillota↑, Bacteroidota↑, Pseudomonadota↓, *Akkermansia*↓, *Bacteroides*↑, *Clostridium*↓, *Escherichia-Shigella*↓.	Not listed.	Not listed.	SCFAs (not tested).	Not listed.	(Lu et al., 2025)
SMI.	C57BL/6 mice. Poly(I:C) (2 mg/kg) i.t., LPS (10 mg/kg) i.p.	Firmicutes/Bacteroidota ratio↑. Chlamydiaceae↓, Lactobacillaceae↑, *Chlamydia abortus*↓.	Not listed.	Not listed.	Not listed.	Lung: NF-κB pathway↓.	(Guo et al., 2025)
YHPG.	ICR mice. 10 × LD50 of H1N1 virus in 50 μL PBS i.t.	Actinobacteria↑, Bacilli↑, *Bifidobacterium*↑, *Blautia*↑, *Dorea*↑, *Escherichia*↓, *Lactobacillus*↑, *Oscillospira*↑.	Not listed.	Colon: injury↓; Claudin-1↑, occludin↑, ZO-1↑.	SCFAs: acetate↑, propionate↑, isobutyrate↑, isovalerate↑.	Lung: GPR43-MAVS-IRF3-IFN-β pathway↑.	(Zeng et al., 2025)
LXHX.	C57BL/6 mice. CLP.	Bacteroidia↑, Gammaproteobacteria↓.	Not listed.	Not listed.	Not listed.	Lung: SIRT1/Nrf2/NF-κB pathway↓.	(Zhong et al., 2025)
SLEL.	SD rats. LPS (5 mg LPS dissolved in 1 mL normal saline, and the volume of injection is 1 mL/kg) i.t.	Actinomycetota↑, Bacillota↑, Bacteroidota↓, Pseudomonadota↑, norank_f:Muribaculaceae↑, *Akkermansia*↑, *Bacteroides*↓, *Lactobacillus*↑.	Not listed.	Not listed.	BAs: primary bile acid biosynthesis (pro-inflammatory bile acid derivatives↓).	Lung: PI3K-Akt pathway↓, MAPK pathway↓.	(Luo et al., 2025)
A novel pectic polysaccharide (GCP-2) was isolated from *Glycyrrhiza uralensis*	C57BL/6 mice. LPS (5 mg/kg body weight in a total volume of 50 μL per mouse) i.t.	Shannon index↑, Chao1 index↑, ACE index↑. Bacteroidetes↑, Firmicutes↓, Proteobacteria↓, Bacteroidaceae↑, *Escherichia-Shigella*↓.	Not listed.	Serum: LPS↓.	Not listed.	Lung: macrophage↓; NF-κB pathway↓, TLR4 pathway↓.	(Yu et al., 2025)

↑ mean increase, and ↓ mean decrease.

##### 4.2.4.1 TCHMs correct gut dysbiosis, preserve intestinal barrier integrity, and inhibit enteric pathogen translocation and DAMPs release

Accumulating evidence indicates that the disruption of the intestinal barrier and subsequent translocation of enteric pathogens and their products are the key triggers of systemic inflammatory storm and pulmonary injury in ALI/ARDS. The efficacy of TCHMs in mitigating this pathological cascade is attributable to two synergistic effects, which align with the fundamental barrier-protective mechanism of probiotics and FMT. On the one hand, TCHMs directly inhibit the overgrowth of intestinal pathogenic bacteria, thereby reducing the source of pro-inflammatory stimuli. Multiple TCM active components and formulations, including sinomenine, Jinhong decoction (JHD), miR-7972 derived from fresh *Rehmanniae Radix*, bear bile powder (BBP) and a novel pectic polysaccharide (GCP-2) from *Glycyrrhiza uralensis*, significantly reduce the relative abundance of intestinal pro-inflammatory pathogens represented by *Citrobacter*, *Enterococcus*, *Enterococcus faecalis*, *Escherichia-Shigella*, *Escherichia coli*, which are the primary sources of PAMPs that drive systemic inflammation (Song et al., 2021; Bao et al., 2023; Qiu et al., 2023; Cheng et al., 2025; Yu et al., 2025). Xuanbai-Chengqi decoction (XBCQ) and naringin dihydrochalcone (NDC) also suppress the expansion of Enterobacteriaceae, a core pathogenic bacterial family closely associated with sepsis and inflammatory injury, further limiting the initiation of inflammatory cascades in ALI/ARDS (Huo et al., 2022; He et al., 2024). On the other hand, TCHMs have been shown to preserve the structural and functional integrity of the intestinal mechanical barrier, thereby impeding the systemic translocation of enteric harmful substances. Sinomenine, JHD, Qingyi decoction (QYD), Yazhicao (*Commelina communis* L., YZC), Xuanfei Baidu decoction (XFBD), Biyuan Tongqiao granules (BYTQ) and *Houttuynia* pectin (HCPM) have been consistently reported to upregulate the expression of intestinal tight junction proteins including ZO-1, occludin, Mucin-1 (Muc-1) and Claudin-1, thus alleviating intestinal pathological injury, and reducing the serum levels of LPS, D-Lactate (D-LA) and diamine oxidase (DAO), the classic biomarkers of intestinal barrier disruption and bacterial translocation (Song et al., 2021; Bao et al., 2023; Wang Z. et al., 2023; Cao et al., 2024; Ni et al., 2025; Yang et al., 2025; Zhu et al., 2025). These effects collectively reduce the systemic release of PAMPs and DAMPs, thereby inhibiting the overactivation of pulmonary inflammatory response at the initiation stage.

##### 4.2.4.2 TCHMs enrich beneficial commensal bacteria, and mediate pulmonary immunomodulation via gut microbiota-derived SCFAs and BAs

Parallel to the core functional mechanism of probiotics and prebiotics, TCHMs exert systemic protective effects by enriching beneficial intestinal commensal bacteria, and further regulating pulmonary immunity via their functional metabolites, mainly SCFAs and BAs. In terms of beneficial bacteria enrichment, multiple TCHMs significantly increase the relative abundance of well-characterized probiotic commensals: Resveratrol (RES), rhubarb, baicalin, astragaloside IV (AS-IV) and Yinhua Pinggan granules (YHPG) markedly upregulate the abundance of *Lactobacillus*, *Lactobacillus reuteri*, which are the core probiotics that maintain intestinal mucosal homeostasis and inhibit pathogen colonization (Alghetaa et al., 2021; Tang et al., 2022; Li et al., 2023; Luo et al., 2024; Zeng et al., 2025). *Lactobacillus* is a potential probiotic that activates the biosynthesis of primary bile acids in the liver and promotes the biotransformation of secondary bile acids in the gut (Hu J. et al., 2023). *Akkermansia* (especially *Akkermansia muciniphila*), a key mucin-degrading commensal that through generation TMAO and SCFAs maintains intestinal barrier function and mucosal immunity (Xiao et al., 2024), is significantly enriched by Kuqin (KQ), QYD, YZC, Jingfang granules (JFG), huperzine A (HupA) and HCPM in ALI/ARDS models (Wang Z. et al., 2023; Xiong et al., 2023; Cao et al., 2024; Su et al., 2024; Xiao et al., 2024; Zhu et al., 2025). In addition, TCHMs also increase the abundance of SCFA-producing bacteria, including SCFAs-producing bacteria (*Akkermansia*, *Alistipes*, *Bacteroides*, *Bifidobacterium*, *Blautia*, *Clostridium*, *Dorea*, *Lactobacillus*, *Oscillospira*, *Parabacteroides*, *Roseburia*), which lay the metabolic foundation for their systemic immunomodulatory effects (Tang et al., 2022; Wang Z. et al., 2023; Xiao et al., 2024; Zeng et al., 2025).

For microbial metabolites, the majority of TCHMs with gut microbiota-modulating effects can elevate the levels of intestinal and circulating SCFAs, the most well-characterized functional metabolites in the gut-lung axis. RES, Gegen Qinlian decoction (GQD), wine-processed radix scutellariae (WRS), total ginsenosides from Panax ginseng stems, baicalein (BAI.), fermented *Rosa roxburghii* juice (RRFJ), HCPM and YHPG have been confirmed to increase the contents of core SCFAs including acetate, propionate, butyrate, isobutyric acid and valeric acid, which can reach the lung through systemic circulation to exert direct immunomodulatory effects (Alghetaa et al., 2021; Li et al., 2022; Ding et al., 2023; Hu T. et al., 2023; Chen et al., 2024; Hu et al., 2024; Zeng et al., 2025; Zhu et al., 2025). Besides, *Cordyceps militaris* solid medium extract (CMME) and sesquiterpene lactones from *Eupatorium lindleyanum* DC. (SLEL) regulate the profile of BAs, including taurocholic acid, cholic acid, deoxycholic acid and chenodeoxycholic acid, and reduce the production of pro-inflammatory BA derivatives, which further modulates pulmonary immune response via the enterohepatic circulation and gut-lung axis (Luo et al., 2025; Wang X. et al., 2025).

##### 4.2.4.3 TCHMs regulate key immune cell circuits and inflammatory signaling pathways in the lung via gut microbiota modulation

The gut microbiota-modulating effects of TCHMs further translate into hierarchical regulation of pulmonary immune cell circuits and inflammatory signaling cascades, which ultimately mitigates the pathological progression of ALI/ARDS. This is consistent with the downstream effector mechanisms of probiotics and FMT. In terms of innate immune cell regulation, which dominates the early stage of ALI/ARDS, multiple TCHMs including miR-7972 derived from fresh *Rehmanniae Radix*, Fuzhengjiedu formula (FZJDF), ethanol extracts from poplar propolis and poplar gum (EEP & EEG), Fuzheng Qushi decoction (FZQS), *Cordyceps militaris* solid medium extract (CMME) and GCP-2 effectively drive the phenotypic switch of alveolar macrophages from pro-inflammatory M1 to anti-inflammatory M2 phenotype, and inhibit the recruitment and overactivation of pulmonary macrophages and neutrophils, the core effector cells that mediate early alveolar injury and inflammatory storm (Qiu et al., 2023; Guo K. et al., 2024; Li Z. et al., 2024; Lu et al., 2024; Wang X. et al., 2025; Yu et al., 2025). JHD also reduces the infiltration of neutrophils and γδT cells in bronchoalveolar lavage fluid (BALF), the key pathogenic subsets that drive alveolar-capillary barrier disruption (Bao et al., 2023). For adaptive immune regulation, RES regulates the activity of cytotoxic T lymphocyte (CTL), NK T cells and Treg cells in the lung and spleen, while rhubarb modulates the balance of Th17/Treg cells, thus maintaining the homeostasis of adaptive immune response and avoiding excessive inflammatory injury (Alghetaa et al., 2021; Tang et al., 2022).

In terms of inflammatory signaling pathways, the NF-κB pathway, the central hub of inflammatory response in ALI/ARDS, is the most common target of TCHMs. XBCQ, baicalin, Yemazhui (*Herba Eupatorii Lindleyani*, HEL), YZC, BBP, EEP & EEG, NDC, FZQS, HupA, Shengmai injection (SMI), Liangxue Huoxue decoction (LXHX) and GCP-2 have been consistently reported to suppress the NF-κB pathway and NLRP3 inflammasome activation, thus reducing the secretion of multiple pro-inflammatory cytokines and chemokines in the lung (Huo et al., 2022; Li et al., 2023; Cao et al., 2024; Guo K. et al., 2024; He et al., 2024; Li R. et al., 2024; Li Z. et al., 2024; Su et al., 2024; Cheng et al., 2025; Guo et al., 2025; Yu et al., 2025; Zhong et al., 2025). In addition, TCHMs regulate multiple non-redundant signaling pathways associated with the gut-lung axis: sinomenine activates the intestinal AhR/Nrf2 pathway to maintain intestinal barrier function (Song et al., 2021); AS-IV and BYTQ modulate the PI3K/AKT/mTOR pathway to alleviate pulmonary inflammatory injury (Luo et al., 2024; Ni et al., 2025); WRS and BAI upregulate the expression of SCFA receptors free fatty acid receptors (FFAR)2 and FFAR3 in lung tissue (Hu T. et al., 2023; Hu et al., 2024), and rhubarb exerts epigenetic regulatory effects via activating HDAC6 activity (Tang et al., 2022), enhancing the pulmonary response to beneficial microbial metabolites.

**Figure 5:**
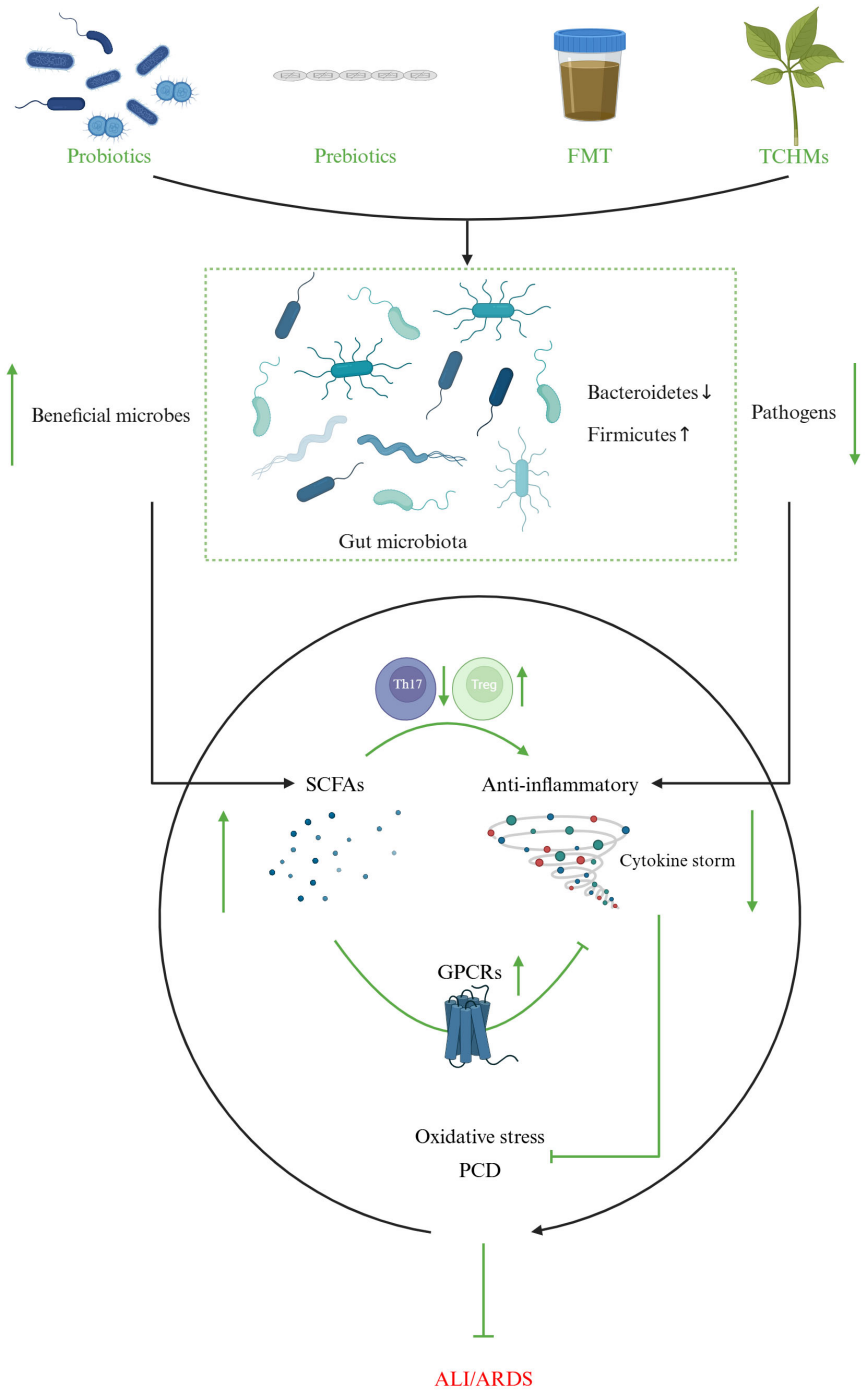
The mechanism of probiotics, prebiotics, FMT, and TCHMs in regulating gut microbiota to mediate the gut-lung axis for the prevention and treatment of ALI/ARDS. GPCRs, G protein-coupled receptors; PCD, programmed cell death; SCFAs, short-chain fatty acids.

Collectively, these findings demonstrate that TCHMs, similar to probiotics, prebiotics, and FMT, exert multi-level protective effects against ALI/ARDS via targeting gut microbiota, regulating microbial metabolite production, and modulating downstream immune cell circuits and inflammatory signaling pathways (**Figure 5**). This provides a solid theoretical basis for the clinical application of TCHMs as microbiota-targeted interventions for ALI/ARDS.

## 5 Translational challenges and future perspectives

Despite significant progress in basic research on the gut-lung axis in ALI/ARDS, the clinical translation of microbiota-targeted therapies still faces multiple core challenges. In this section, we systematically analyze these translational challenges, and propose future research directions based on multi-omics integration and artificial intelligence technology, with the aim of promoting the transformation of basic research into clinical practice.

### 5.1 Core translational challenges

#### 5.1.1 Heterogeneity of ARDS patients

ARDS is a heterogeneous syndrome with multiple etiologies, and the gut microbiota characteristics and immune status of patients with different etiologies (sepsis, trauma, viral infection, etc.) are significantly different. The “one-size-fits-all” microbiota-targeted intervention strategy is incapable of meeting the diverse needs of patients, which is the fundamental reason for the inconsistent efficacy of clinical trials.

#### 5.1.2 Gap between animal models and clinical reality

The preponderance of extant experimental evidence derives from SPF-grade rodent models; however, it is imperative to acknowledge the fundamental difference in the composition of the gut microbiota between rodents and humans. Furthermore, the use of standardized animal modeling has been shown to fall short in its replication of the complex comorbidities, drug exposure, and dynamic disease course characteristic of critically ill patients. This discrepancy results in a significant gap between experimental findings and their translation into clinical practice.

#### 5.1.3 Safety risks of microbiota-targeted interventions

Critically ill patients frequently exhibit impaired intestinal barrier function and immunosuppression. Probiotic and FMT interventions carry the potential risks of bacteremia and iatrogenic infection. The dearth of standardized operation protocols and safety evaluation systems further curtails the clinical implementation of these strategies. In contrast to probiotics or prebiotics, which have well-defined active components, the multi-component nature of TCHMs complicates the distinction between direct pharmacological effects and gut microbiota-mediated indirect effects.

#### 5.1.4 Lack of validated biomarkers

A significant impediment to the delivery of precise treatment regimens pertains to the dearth of validated biomarkers capable of predicting the efficacy of microbiota-targeted interventions, encompassing probiotics, prebiotics, FMT, and TCHMs. The absence of such biomarkers hinders the ability to stratify patients according to their likelihood of responding positively to these therapeutic modalities, constituting a crucial bottleneck in the pursuit of precision treatment.

### 5.2 Future research directions

#### 5.2.1 Construct a clear conceptual framework and deepen mechanistic research

Subsequent research endeavors should prioritize the investigation of the fundamental scientific query: “How do gut-derived microbial signals influence pulmonary innate immune responses during ALI/ARDS?” This inquiry aims to elucidate the comparative contributions of disparate pathways (direct PAMP activation versus metabolite-mediated regulation) across various etiologies and stages of ARDS. The utilization of single-cell multi-omics technology is imperative for the analysis of cell-type-specific regulatory mechanisms of gut-derived signals in the lung, thereby facilitating a more profound comprehension of the gut-lung axis (Wu et al., 2024; Tong et al., 2026; Wang et al., 2026).

#### 5.2.2 Develop multi-omics and AI-based precision patient stratification

The integration of metagenomics, metabolomics, transcriptomics, and immunophenotyping data is essential for the construction of a multidimensional biomarker system for patients with ARDS. The implementation of machine learning algorithms is crucial for the identification of microbiota-associated ARDS phenotypes and the stratification of patients who are most likely to benefit from microbiota-targeted therapies. To illustrate, machine learning models have the potential to integrate host transcriptomic and microbiome data in ARDS cohorts. This integration would facilitate the identification of key microbiota-derived metabolites driving lung inflammation. Furthermore, such models could predict the efficacy of SCFAs supplementation, probiotic intervention, and TCHMs.

#### 5.2.3 Optimize microbiota-targeted intervention strategies and conduct high-quality clinical trials

The development of colon-targeted delivery systems for SCFAs and prebiotics is crucial for enhancing their local bioavailability within the colon. The construction of engineered probiotics with precise therapeutic functions is paramount for mitigating safety concerns. The development of gut-targeted delivery systems for TCHMs active ingredients, such as pH-sensitive microcapsules for baicalin, is essential. Conducting large-scale, multicenter, randomized, double-blind, placebo-controlled clinical trials is imperative to substantiate the efficacy and safety of microbiota-targeted therapies in patients with ALI/ARDS. Strict patient stratification based on biomarkers is necessary for these trials.

#### 5.2.4 Explore synergistic TCHMs-combination strategies

In light of TCHMs’ multi-target characteristics, the exploration of combinations of TCHMs with probiotics/prebiotics to enhance microbiota modulation is a promising avenue for further research. For instance, a combination of baicalin (which has been demonstrated to promote the growth of *Lactobacillus*) with inulin (a prebiotic) has been shown to produce a synergistic effect, resulting in a significant increase in gut SCFAs and a concomitant alleviation of pulmonary inflammation. These combinations can leverage TCHMs principles that optimize the gut microenvironment and the direct microbiota-restoring effects of probiotics. This addresses the heterogeneity of individuals when using single-intervention strategies.

## 6 Conclusion

The gut-lung axis has emerged as a core research frontier in the pathogenesis of ALI/ARDS. This has fundamentally changed our understanding of the disease from a lung-centric view to a systemic regulatory view. Accumulating evidence has confirmed that gut microbiota dysbiosis is not only a secondary consequence of critical illness, but also a key causal driver of the onset and progression of ALI/ARDS. The gut microbiota has been shown to regulate pulmonary immune responses via two core pathways: direct immune activation by microbial structural components, and remote immunomodulation by gut microbial metabolites. A series of highly conserved immune cell circuits (including ILCs, AMs, neutrophils, DCs, and adaptive immune cells) function as downstream effectors of these pathways. A plethora of studies have demonstrated the efficacy of microbiota-targeted therapeutic strategies, including probiotics, prebiotics, FMT, and TCHMs, in preclinical models. However, the translation of these strategies into clinical practice remains challenging due to various factors.

Future research should prioritize the development of a comprehensive conceptual framework, the advancement of mechanistic research in the field of gut-lung communication, the integration of multi-omics and AI to facilitate precision patient stratification, the execution of high-quality clinical trials, and the investigation of the synergistic effects of multi-organ axes. As research progresses, the gut-lung axis is poised to offer novel theoretical frameworks and therapeutic targets for the prevention and treatment of ALI/ARDS, thereby enhancing the prognosis of critically ill patients.

## Glossary

acetyl-CoA: acetyl-coenzyme A
AECs: alveolar epithelial cells
AhR: aryl hydrocarbon receptor
AI: artificial intelligence
AKT: protein kinase B
ALI: acute lung injury
AMs: alveolar macrophages
AP: acute pancreatitis
APCs: antigen-presenting cells
ARDS: acute respiratory distress syndrome
AS-IV: astragaloside IV
ATP: adenosine triphosphate
BAI: baicalein
BALF: bronchoalveolar lavage fluid
BAs: bile acids
BBP: bear bile powder
BLM: bleomycin
BYTQ: Biyuan Tongqiao granules
CA: cholic acid
CDCA: chenodeoxycholic acid
cfDNA: circulating free DNA
CLP: cecal ligation and puncture
CMME: *Cordyceps militaris* solid medium extract
Covid-19: coronavirus disease 2019
CTL: cytotoxic T lymphocyte
CYP7A1: cholesterol 7α-hydroxylase
CYP27A1: sterol 27-hydroxylase
DAMPs: damage-associated molecular patterns
DCA: deoxycholic acid
DCs: dendritic cells
DYY: Dayuan Yin
EBEE: *Elsholtzia bodinieri Vaniot* ethyl acetate extract
eCIRP: extracellular cold-induced RNA-binding protein
EEP: ethanol extract from poplar propolis
EEG: ethanol extract from poplar tree gum
ERK: extracellular signal-regulated kinase
FFAR: free fatty acid receptors
FMT: fecal microbiota transplantation
FOS: fructooligosaccharides
FZJDF: Fuzhengjiedu formula
FZQS: Fuzheng-Qushi decoction
GCP-2: *Glycyrrhiza* polysaccharide fraction 2
GPCRs: G protein-coupled receptors
GQD: Ge-Gen-Qin-Lian decoction
HCP: *Houttuynia cordata* polysaccharides
HDACs: histone deacetylases
HEL: *Herba Eupatorii Lindleyani*
HMGB1: high mobility group box protein B1
HO-1: heme oxygenase 1
HSPs: heat shock proteins
HupA: huperzine A
ICU: intensive care unit
IFN: interferon
IL: interleukin
ILCs: innate lymphoid cells
ILC2s: type 2 ILCs
ILC3s: type 3 ILCs
i.n.: intranasal instillation
i.p.: intraperitoneal injection
IRF: interferon regulatory factor
i.t.: intratracheal instillation
i.v.: intravenous injection
JAK: Janus kinase
JFG: Jingfang granules
JHD: Jinhong decoction
JNK: c-Jun N-terminal kinase
KQ: kuqin
LBP: LPS binding protein
LCA: lithocholic acid
LPS: lipopolysaccharide
LRP: *Lycium ruthenicum* polysaccharide
LXHX: Liangxue Huoxue decoction
MAIT: mucosal-associated invariant T
MALT: mucosa-associated lymphoid tissue
MAPK: mitogen-activated protein kinase
MAVS: mitochondrial antiviral signaling protein
MRSA: methicillin-resistant *Staphylococcus aureus*
mTOR: mammalian target of rapamycin
NDC: naringin dihydrochalcone
NETs: neutrophil extracellular traps
NF-κB: nuclear factor kappa B
NK: natural killer
NLRP3: NOD-like receptor thermal protein domain associated protein 3
NLRs: NOD-like receptors
Nrf2: nuclear factor erythroid 2-related factor-2
OTUs: operational taxonomic units
OXPHOS: mitochondrial oxidative phosphorylation
PAMPs: pathogen-associated molecular patterns
PCD: programmed cell death
PGN: peptidoglycan
PI3K: phosphatidyqinositol‐3 kinase
PRRs: pattern recognition receptors
QYD: Qingyi decoction
RCT: randomized controlled trial
RES: resveratrol
RRFJ: fermented *Rosa roxburghii* juice
SARS-CoV-2: severe acute respiratory syndrome coronavirus 2
SCFAs: short-chain fatty acids
SD: Sprague Dawley
SEB: staphylococcal enterotoxin‐B
Sfb: segmented filamentous bacteria
SIgA: secretory IgA
SIRT1: silent information regulator 1
SLEL: sesquiterpene lactones from *Eupatorium lindleyanum* DC.
SMI: Shenmai injection
sp.: species
S1P: sphingosine-1-phosphate
STAT: signal transducer and activator of transcription
SUCNR1: succinate receptor 1
TCHMs: traditional Chinese herbal medicines
TFXF: Tongfu Xiefei Guanchang solution
TGF: transforming growth factor
TGR5: G protein-coupled bile acid receptor 1
Th17: T helper cell 17
TLRs: Toll-like receptors
TMAO: Trimethylamine N-oxide
TNF: tumor necrosis factor
Treg: regulatory T cells
VAP: ventilator-associated pneumonia
WRS: rice wine process radix scutellariae
XBCQ: Xuanbai–Chengqi decoction
XFBD: Xuanfei Baidu decoction
YHPG: Yinhua Pinggan granules
YZC: Commelina *communis* L
ZO-1: zonula occludens-1.
